# Key genes in a “Galloylation-Degalloylation cycle” controlling the synthesis of hydrolyzable tannins in strawberry plants

**DOI:** 10.1093/hr/uhae350

**Published:** 2024-12-16

**Authors:** Lingjie Zhang, Rui Li, Maohao Wang, Qiaomei Zhao, Yifan Chen, Yipeng Huang, Yajun Liu, Xiaolan Jiang, Nana Wang, Tao Xia, Liping Gao

**Affiliations:** School of Life Science, Anhui Agricultural University, West 130 Changjiang Road, Hefei 230036, Anhui, China; School of Life Science, Anhui Agricultural University, West 130 Changjiang Road, Hefei 230036, Anhui, China; School of Life Science, Anhui Agricultural University, West 130 Changjiang Road, Hefei 230036, Anhui, China; School of Life Science, Anhui Agricultural University, West 130 Changjiang Road, Hefei 230036, Anhui, China; State Key Laboratory of Tea Plant Biology and Utilization/Key Laboratory of Tea Biology and Tea Processing of Ministry of Agriculture/Anhui Provincial Laboratory of Tea Plant Biology and Utilization, Anhui Agricultural University, West 130 Changjiang Road, Hefei 230036, Anhui, China; State Key Laboratory of Tea Plant Biology and Utilization/Key Laboratory of Tea Biology and Tea Processing of Ministry of Agriculture/Anhui Provincial Laboratory of Tea Plant Biology and Utilization, Anhui Agricultural University, West 130 Changjiang Road, Hefei 230036, Anhui, China; School of Life Science, Anhui Agricultural University, West 130 Changjiang Road, Hefei 230036, Anhui, China; State Key Laboratory of Tea Plant Biology and Utilization/Key Laboratory of Tea Biology and Tea Processing of Ministry of Agriculture/Anhui Provincial Laboratory of Tea Plant Biology and Utilization, Anhui Agricultural University, West 130 Changjiang Road, Hefei 230036, Anhui, China; School of Life Science, Anhui Agricultural University, West 130 Changjiang Road, Hefei 230036, Anhui, China; State Key Laboratory of Tea Plant Biology and Utilization/Key Laboratory of Tea Biology and Tea Processing of Ministry of Agriculture/Anhui Provincial Laboratory of Tea Plant Biology and Utilization, Anhui Agricultural University, West 130 Changjiang Road, Hefei 230036, Anhui, China; School of Life Science, Anhui Agricultural University, West 130 Changjiang Road, Hefei 230036, Anhui, China

## Abstract

Strawberry fruits, known for their excellent taste and potential health benefits, are particularly valued for their rich content of hydrolyzable tannins (HTs). These compounds play key roles in regulating growth and development. However, the molecular mechanisms underlying HT synthesis in plants remains poorly elucidated. In this study, based on a correlation analysis between the transcriptome and metabolome of HTs, galloyl glucosyltransferase (UGT84A22), serine carboxypeptidase-like acyltransferases (SCPL-ATs), and carboxylesterases (CXEs) were screened. Furthermore, *in vitro* enzymatic assays confirmed that FaSCPL3-1 acted as a hydrolyzable tannins synthase (HTS), catalyzing the continuous galloylation of glucose to form simple gallotannins (GTs). Additionally, FaCXE1/FaCXE3/FaCXE7 catalyzed the degalloylation of simple GTs and ellagitannins (ETs), and FaUGT84A22 catalyzed the glycosylation of gallic acid (GA) to produce 1-*O*-β-glucogallin (βG), a galloyl donor. Moreover, in *FvSCPL3*-*1*-RNAi transgenic strawberry plants, the contents of simple GT and some ET compounds were reduced, whereas, in *FaCXE7* overexpressing strawberry plants, these compounds were increased. These enzymes constituted a biosynthetic pathway of galloyl derivatives, termed the “galloylation-degalloylation cycle” (G-DG cycle). Notably, the overexpression of *FaCXE7* in strawberry plants not only promoted HT synthesis but also interfered with plant growth and development by reducing lignin biosynthesis. These findings offer new insights into the mechanisms of HT accumulation in plants, contributing to improving the quality of berry fruits quality and enhancing plant resistance.

## Introduction

Hydrolyzable tannins (HTs) are a group of plant polyphenols with molecular weights greater than 500. They can be divided into two categories: gallotannins (GTs) and ellagitannins (ETs) [1, 2]. Under the action of 1-*O*-β-glucogallin (βG, compound 1 in Supplementary Data Fig. S1) dependent galloylation of glucose, simple GTs with two to five galloyl groups in a strict positional order are synthesized, including 1,6-digalloylglucose (DGG, compound 2 in Supplementary Data Fig. S1), 1,3,6-trigalloylglucose (1,3,6-TGG, compound 3 in Supplementary Data Fig. S1), 1,2,3,6-tetragalloylglucose (1,2,3,6-TeGG, compound 4 in Supplementary Data Fig. S1), and 1,2,3,4,6-pentagalloylglucose (1,2,3,4,6-PGG, compound 5 in Supplementary Data Fig. S1). PGG serves as a precursor in the biosynthesis of GTs and ETs. Complex GTs, characterized by meta-depsides, are formed by adding additional galloyl groups to the PGG molecule, with βG as an acyl donor [3]. ETs are monomeric or polymerized compounds containing hexahydroxydiphenoyl (HHDP) groups, which are produced through laccase-catalyzed oxidation of two adjacent galloyl groups in PGG [4, 5]. Upon hydrolysis, GTs and ETs release gallic acid (GA, compound 6 in Supplementary Data Fig. S1) and ellagic acid (EA, compound 7 Supplementary Data in Fig. S1), respectively [2, 6]. Structurally, EA is a dilactone of hexahydroxydiphenic acid generated from the oxidative dimerization of GA. EA glycosides are then synthesized through the hydrolysis of ETs and subsequent glycosylation.

Strawberry fruits are abundant in antioxidant substances, such as ascorbic acid, EA, EA glycosides, ETs, and anthocyanins [7, 8]. A variety of EA glycosides and ETs have been identified in strawberry fruits and leaves using detection techniques combined with liquid chromatography and mass spectrometry [9, 10]. Notable compounds include strictinin (compound 8 in Supplementary Data Fig. S1), pedunculagin (compound 9 in Supplementary Data Fig. S1), casuarictin (compound 10 in Supplementary Data Fig. S1), sanguiin H-6 (compound 11 in Supplementary Data Fig. S1), and ellagic acid deoxyhexose (compound 12 in Supplementary Data Fig. S1).

ETs and EA from strawberries, pomegranates, and walnuts are metabolized by intestinal microorganisms in the human body to produce bioactive compounds called urolithins [11]. Furthermore, an ET (HeT, 1-*O*-galloyl-2,3; 4,6-bis-HHDP-β-D-glucopyranose), derived from strawberry leaves, has been identified as a potential plant defense molecule capable of inducing pathogen resistance in strawberry and lemon plants [12]. Strawberries possess excellent taste and potential health benefits [13, 14], garnering significant attention in their quality improvement, regulation of growth and development, and cultivation management [15, 16]. Therefore, it is essential to explore the biosynthesis and regulation of HTs in plants rich in HTs, such as strawberries.

While the synthesis and regulatory mechanisms of condensed tannins have been extensively studied, research on HT synthesis remains in the early stages [17]. The synthesis and metabolism of galloylglucose esters and flavan-3-ol gallates involve the transfer (galloylation) and hydrolysis (degalloylation) of galloyl groups. The galloylation and degalloylation of simple HTs in oak (*Quercus rubur* and *Q. rubra*) and sumac (*Rhus typhina*) have been identified using enzymatic methods, suggesting that multiple enzymes are involved in these processes [18]. However, the specific genes responsible for these reactions remain unidentified.

**Figure 1:**
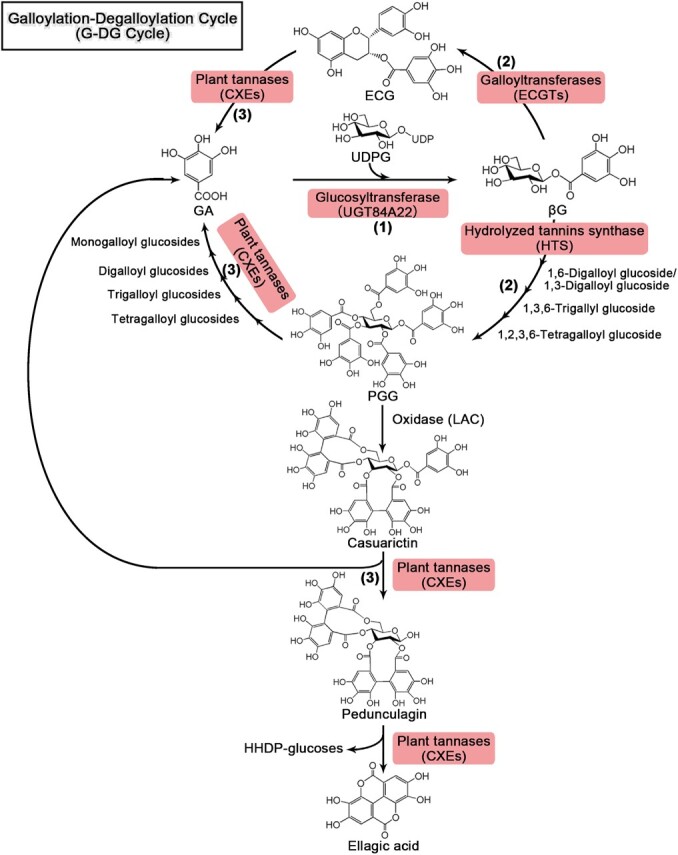
Pathway of the “galloylation-degalloylation cycle” (G-DG cycle) in plants. (1) glycosylation; (2) galloylation; (3) degalloylation

Significant progress has been made in understanding the galloylation and degalloylation mechanisms of galloyl catechins (Fig. 1), including the identification of genes encoding galloyl glucosyltransferase (UGT84A22) [19], galloyltransferases (serine carboxypeptidase-like acyltransferases, SCPL-ATs) [20, 21], and plant tannases or carboxylesterases (TAs/CXEs) [22, 23]. Two paralogous SCPL-AT genes are involved in the galloylation of flavan-3-ols in tea plants: CsSCPL4 (acting as a catalytic acyltransferase) and CsSCPL5 (functioning as a noncatalytic companion protein of CsSCPL4) [21]. Homologous TAs, members of the CXEs family, can break ester bonds within flavan-3-ol gallates and simple HTs, such as PGG, releasing GA [22]. A recent study has indicated that CsTA is a hydrolase with promiscuous acyltransferase activity [23]. The results of the transient overexpression and silencing experiments of *TA* genes in these studies demonstrated a positive correlation between *TA* gene expression levels and the content of ETs in strawberries, as well as GTs and flavan-3-ol gallates in tea plants [22, 23].

Based on these findings, this study speculated the existence of a “galloylation-degalloylation cycle” (G-DG cycle) in plants rich in tannins (Fig. 1). In this cycle, TAs accelerates the utilization of GA through hydroxylase and promiscuous acyltransferase activity, promoting the accumulation of ETs in strawberries and GTs, and flavan-3-ol gallates in tea plants. Additionally, the increased turnover of this cycle may negatively regulate immediate metabolic neighborhoods, such as the lignin and flavonoid pathways, by diverting carbohydrates. However, since woody plants, including tea plants and oaks, are difficult to genetically modify, the true functions of the genes involved in galloylation and degalloylation have yet to be fully verified in these plants.

This study identified *UGT84A22*, *SCPL-AT*, and *CXE* genes as key regulators of HT accumulation in strawberry plants. *In vitro* enzymatic assays revealed that FaSCPL3-1 functions as a hydrolyzable tannin synthase (HTS), catalyzing the continuous galloylation of glucose to produce simple GTs. FaCXE1/FaCXE3/FaCXE7 were confirmed as TA enzymes responsible for the degalloylation of simple GTs and ETs. FaUGT84A22-1 catalyzed the glycosylation of GA to form βG. Additionally, stable genetic transformation of strawberry plants via *FvSCPL3*-*1*-RNAi and overexpression of *FaCXE7* demonstrated their roles in HT accumulation*.* Moreover, *FaCXE7* not only promoted HT synthesis but also disrupted plant growth and development by inhibiting lignin biosynthesis.

## Results

### Detection of HTs in strawberry plants

Strawberries are rich in galloyl phenolic compounds, including galloyl catechins, GTs and ETs. Galloyl catechins and GTs feature a central glucose or catechins linked to one or more GA units. ETs consist of HHDP units formed by the oxidative linkage of galloyl groups and glucose. EA is a dilactone of hexahydroxydiphenic acid resulting from the dimerization of GA. EA glycosides are primarily formed by the glycosylation of ET hydrolysates.

**Figure 2:**
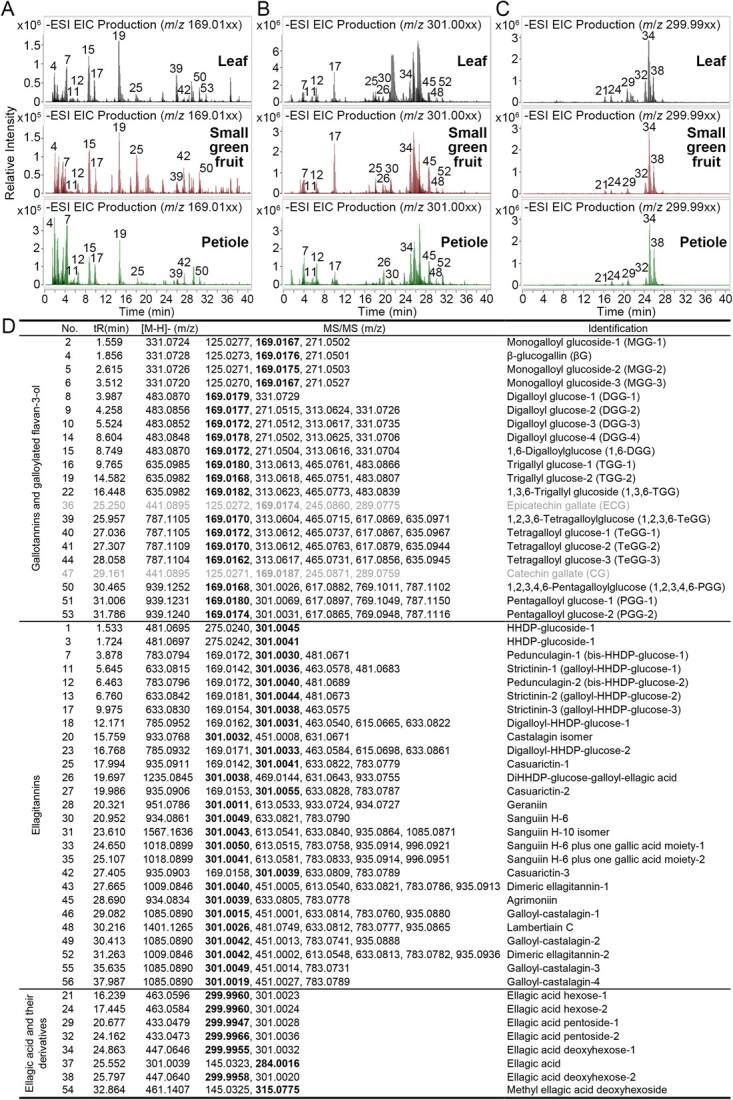
Identification of galloyl phenolic compounds in various strawberry tissues by Q-TOF-UPLC/MS. A-C, EIC diagrams of galloyl phenolic compounds, with characteristic fragment ions at *m*/*z* 169.01xx (GA group) (A), *m*/*z* 300.99xx (HHDP group), and *m*/*z* 299.99xx (EA group) (C) in extracts from leaves, fruits, and petioles. D, Identification and comparative analysis of galloyl compounds in extracts from different strawberry tissues. Galloyl phenolic compounds include 19 gallotannins (GTs) and 2 galloylated flavan-3-ol ), 27 ellagitannins (ETs) and 8 ellagic acid (EA) and their derivatives.

Based on standards and literature data [9, 22, 24, 25], galloyl phenolic compounds, including flavan-3-ol gallates, GTs, ETs, and EA glycosides, were qualitatively identified in various organs of octoploid strawberry plants. This identification was based on retention time (*t_R_*) and the mass-to-charge ratio (*m*/*z*) of charged parent ions and fragment ions in the extracted ion chromatograms (EIC), using characteristic fragment ions (*m*/*z* 169.00 for GA, 301.00 for HHDP, and 299.99 for EA) as extraction ions in the negative mode of UPLC coupled with quadrupole time-of-flight mass spectrometry (Q-TOF-UPLC/MS) (Fig. 2A-C and Supplementary Data Fig. S2). Among the 56 identified galloyl compounds, only two were flavan-3-ol gallates (peak 36 and peak 47, i.e. epicatechin gallate and catechin gallate), and the remaining 54 were HTs (Fig. 2D). These HTs were further categorized into three types: simple GTs, monomeric and dimeric ETs, and EA and its glycosides. Oligomeric HT derivatives were not detected. Quantitative analysis of galloyl phenolic compounds in different organs and leaves at various developmental stages was performed based on the peak area values of characteristic parent and fragment ion pairs, as determined by Q-TOF-UPLC/MS (Supplementary Data Tables S1 and S2).

By comparing the retention time and fragmentation patterns to standards and literature data, a total of 19 GTs with characteristic fragment ions at *m*/*z* 169.01xx were detected in various strawberry organs (Fig. 2D and Supplementary Data Fig. S2). Based on these standards, some simple GTs with diverse numbers of GA groups were accurately identified, including peak 4 (βG, *m*/*z* 331.0728), peak 15 (1,6-DGG, *m*/*z* 483.0870), peak 22 (1,3,6-TGG, *m*/*z* 635.0982), peak 39 (1,2,3,6-TeGG, *m*/*z* 787.1105), and peak 50 (1,2,3,4,6-PGG, *m*/*z* 939.1252). All other simple GTs were identified as isomers of these compounds. The total content of simple GTs in the leaves, flowers, petioles, small green fruits, roots, white fruits, and red fruits decreased sequentially (Supplementary Data Table S1). The concentrations of these compounds in white and red fruits were almost undetectable. Furthermore, as the leaves developed, the levels of GTs gradually dropped (Supplementary Data Table S2). TGG-2 (peak 19) and 1,2,3,6-TeGG (peak 39) were the dominant GTs across various organs.

ETs are significant natural compounds in strawberry plants, playing a role in the plant’s defense against pathogens [12]. A total of 27 monomeric and dimeric ETs with the characteristic fragment ion at *m*/*z* 301.00, corresponding to the HHDP group, were detected in various organs. These included isomers of strictinin, casuarictin, sanguiin H-6, pedunculagin, and castalagin (Fig. 2D and Supplementary Data Fig. S2). Based on relative content calculated using cumulative peak area, ETs were most abundant in tender leaves, flowers, and petioles, and their content was low in roots and white and red fruits (Supplementary Data Tables S1 and S2). This distribution may be related to the plant’s defense mechanism. Two pedunculagins with parent ion *m*/*z* 783.079x (peaks 7 and 12), casuarictin-1 with parent ion *m*/*z* 935.0911 (peak 25), strictinin-3 with parent ion *m*/*z* 633.0830 (peak 17), and agrimoniin with parent ion *m*/*z* 934.0834 (peak 45) were the dominant ETs.

Some compounds with parent ions at *m*/*z* 433.047x, *m*/*z* 447.0646, *m*/*z* 463.05xx, and *m*/*z* 461.1407 were identified as EA glycosides with characteristic fragment ions at *m*/*z* 299.99 or *m*/*z* 301.00 (Fig. 2D and Supplementary Data Fig. S2). Similar to simple GTs and ETs, the content of EA glycosides in flowers, leaves, and petioles was significantly higher than that in roots and mature fruits (Supplementary Data Table S1). Among the EA glycosides, ellagic acid deoxyhexoses with parent ions *m*/*z* 447.0646 (peaks 34 and 38) were the dominant compounds in various strawberry organs. These findings suggest that HTs predominantly accumulate in the leaves and flowers of strawberry plants.

### Correlation analysis of gene expression related to HT synthesis and metabolite accumulation

**Figure 3:**
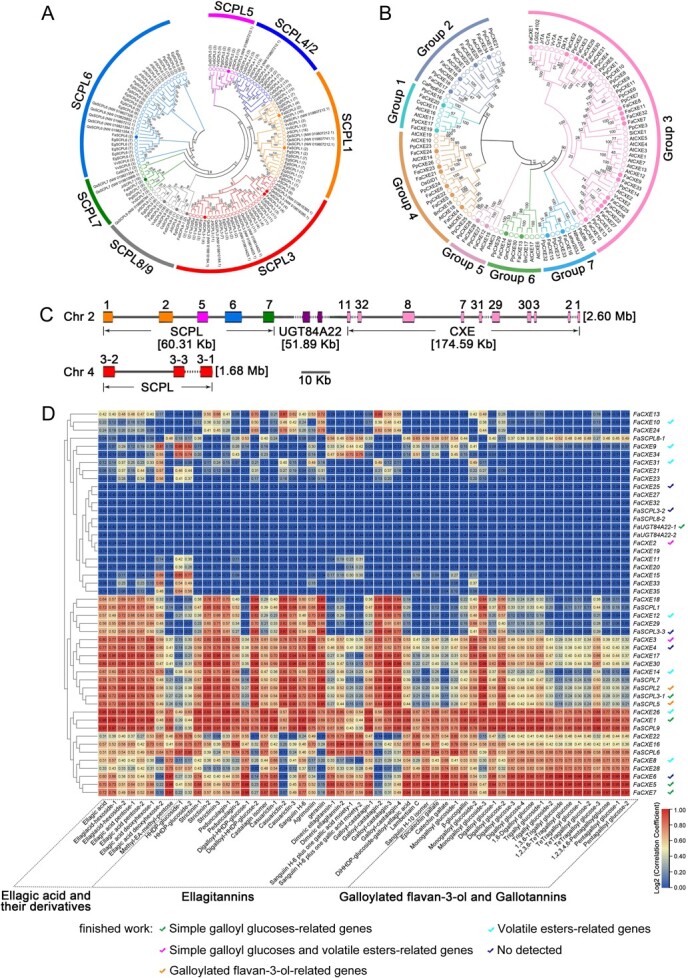
Correlation analysis between expression of phenolic compound-related genes and accumulation of galloyl phenolic compounds in strawberry plants. A and B, Phylogenetic analysis of the SCPL-AT and CXE families. Phylogenetic trees were generated using the neighbor-joining method in MEGA 5.0, with 1000 bootstrap replicates. The accession numbers of SCPL-ATs and CXEs are listed in Supplementary Data Table S3. SCPL-AT proteins were sourced from *Camellia Sinensis* (*Cs*), *Vitis vinifera* (*Vv*), *Punica granatum* (*Pg*), *Eucalyptus grandis* (*Eg*), *Quercus suber* (*Qs*), and *Juglans regia* (*Jr*). CXE proteins were obtained from *A. thaliana* (*At*), *Prunus persica* (*Pp*), and other plants (their functions have been identified in earlier studies) (Supplementary Data Table S3c). SCPL-ATs and CXEs from strawberry and other plants are marked with dots and circles, respectively. C, Chromosomal distribution of *FaSCPL-ATs*, *FaUGT84A22s*, and *FaCXEs* in the strawberry genome. D, Correlation analysis between the expression levels of *FaSCPL-ATs*, *FaUGT84A22s*, and *FaCXEs* and the content of galloyl phenolic compounds in strawberry plants. The checkmarks represent the detected enzymatic activities of some CXEs. The colored checkmarks denote that different CXE enzymatic activities.

Previous studies have identified several genes involved in the synthesis and metabolism of galloyl phenolic compounds, including *UGT84A22*, *SCPL-AT*, and *CXE* (*TA*) genes [19–23]. The sequence information of these three gene families was retrieved from the genome databases of several plants for comparative analysis, including *Fragaria vesca* (*Fv*) and tannin-rich plants, such as *Camellia sinensis* (*Cs*), *Vitis vinifera* (*Vv*), *Punica granatum* (*Pg*), *Eucalyptus grandis* (*Eg*), *Quercus suber* (*Qs*), and *Juglans regia* (*Jr*). A total of 11 *SCPL-AT* genes, 17 *CXE* members (belonging to the third subfamily), and two *UGT84A22* genes were screened in the strawberry genome database (Fig. 3A and B) (Supplementary Data Table S3a).

The gene cluster responsible for flavan-3-ol galloylation (comprising five *FaSCPL-AT* genes and two *FaUGT84A22* orthologs) and a *CXE* gene cluster (containing 10 *FaCXE* members) was mapped to chromosome 2 of the strawberry genome (Fig. 3C). Among the *FaSCPL-AT* genes, *FaSCPL1, FaSCPL2,* and *FaSCPL5* are homologous to *CsSCPL4* and *CsSCPL5*, which have been proven to be involved in the galloylation of flavanols in tea plants. Within the *CXE* gene cluster, *FaCXE1* (also known as *FaTA*) has been implicated in the hydrolysis of galloylated flavan-3-ols and HTs [22], whereas *FaCXE2* and *FaCXE3* are involved in the hydrolysis of volatile acetate esters [26]. Additionally, three *FaSCPL3* genes were located on chromosome 4, with two of them closely linked to their adjacent positions. When comparing the genome data of octoploid strawberry with diploid strawberry (*F. vesca*), the number of *SCPL-ATs* and *CXEs* in the octoploid strawberry genome was nearly quadrupled. However, the number of these genes in strawberry plants was comparable to that in other plants (Supplementary Data Table S3b).

The expression levels of these genes were determined through RNA-seq analysis of leaves, petioles, flowers, small green fruits, white fruits, red fruits, and roots from Benihoppe strawberry plants. Principal component analysis (PCA) revealed clear separation between organ samples, indicating that each organ has a distinct gene expression profile (Supplementary Data Fig. S3 and Table S4).

Figure S4 presents heatmap analysis of 35 *FaCXEs*, 2 *FaUGT84A22s*, and 11 *FaSCPL-ATs*. The expression levels of *FaUGT84A22*-*1* and *FaCXE24* are significantly higher than those of other genes in various tissues and organs, indicating they are constitutively expressed. In contrast, some genes, such as *FaCXE10*, *FaCXE11*, *FaCXE13*, *FaCXE15*, *FaCXE18*, *FaCXE23*, *FaCXE30*, *FaCXE32*, and *FaCXE33*, exhibited low expression levels across various tissues. The expression levels of *FaCXE1*, *FaCXE3*, *FaCXE4*, *FaCXE7*, *FaCXE9*, *FaSCPL1*, *FaSCPL2*, *FaSCPL5*, *FaSCPL3*-*1*, and *FaSCPL7* genes were much higher in leaves and flowers than in other organs. In addition, *FaCXE21* showed high expression in roots and flowers, and *FaCXE27* and *FaUGT84A22*-*2* were highly expressed in mature red fruits.

A clustered heatmap of the correlation between the content of galloyl compounds detected by UPLC-Q-TOF-MS and the expression of *FaUGT84A22s*, *FaSCPL-ATs*, and *FaCXEs* based on RNA-seq data from strawberry plants was generated (Fig. 3D). The results showed that the gene expression of *FaCXE1*, *FaCXE3*, *FaCXE4*, *FaCXE5*, *FaCXE6*, *FaCXE7*, *FaCXE8*, *FaCXE16*, *FaCXE22*, *FaCXE26*, *FaCXE17*, *FaCXE28*, *FaCXE30*, *FaSCPL1*, *FaSCPL2*, *FaSCPL3*-*1*, *FaSCPL5*, *FaSCPL6*, and *FaSCPL9* was highly correlated with the accumulation of simple GTs, ETs, and EA glycoside compounds. Additionally, negative correlations were observed between some genes and the accumulation of HTs and other substances, such as *FaCXE2*, *FaCXE19*, *FaCXE25*, *FaCXE27*, *FaCXE32*, *FaSCPL3*-*2*, *FaSCPL8*-*2*, and *FaUGT84A22s*. These findings suggest that some *FaCXE* and *FaSCPL* genes may be involved in the accumulation of HTs.

### Enzymatic validation of FaUGT84A22-1, FaSCPL-ATs, and FaCXEs

Based on the correlation analysis and gene expression profiles (Supplementary Data Table S4b), one *FaUGT84A22*, five *FaSCPL-ATs*, and 15 *FaCXE*s were selected for functional verification. Strawberry transcriptome data were used to design specific upstream and downstream primers for gene cloning (Supplementary Data Table S5). The open reading frames (ORFs) of the above genes were successfully cloned via high-fidelity polymerase.

**Figure 4:**
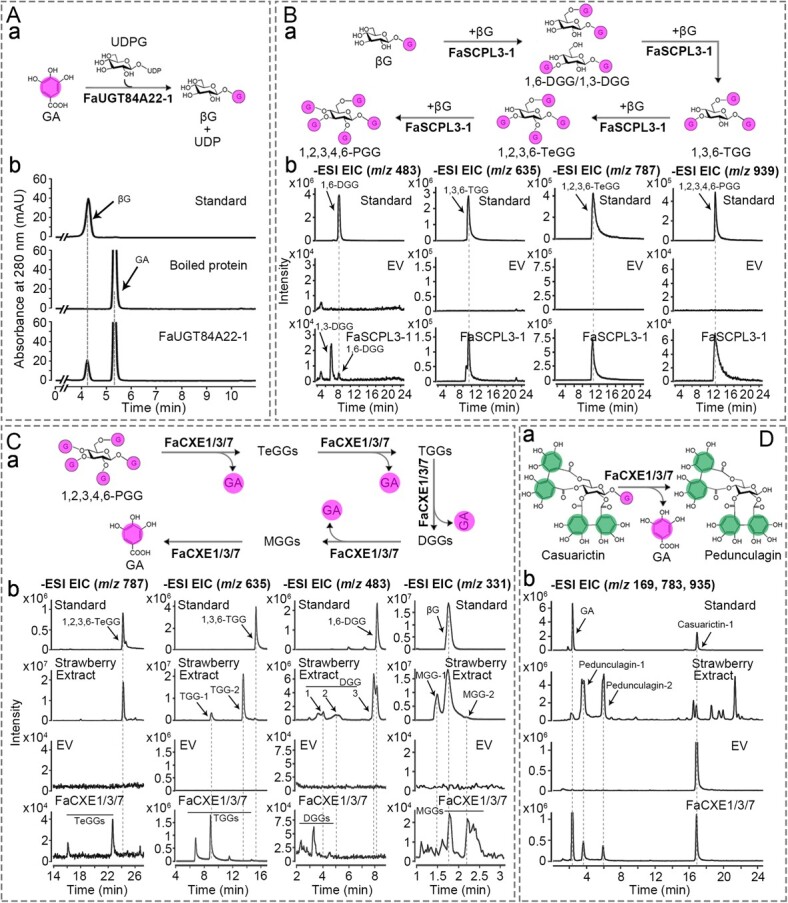
Enzymatic activity analysis of recombinant FaUGT84A22-1, FaSCPL3-1, and FaCXE proteins. A, UPLC analysis of enzymatic products of FaUGT84A22-1 purified from *E. coli* using GA and UDPG as substrates. a, Diagram of FaUGT84A22-1 catalyzing gallic acid (GA) and UDPG to βG. b, Enzyme activity detection of FaUGT84A22-1 in vitro. B, FaSCPL3-1 catalyzes the four consecutive biosynthesis steps from βG to 1,2,3,4,6-pentagalloylglucose (1,2,3,4,6-PGG). a, Diagram of continuous galloylation catalyzed by FaSCPL3-1 from βG to PGG. b, Detection of enzymatic reaction products using βG only, βG and 1,6-digalloylglucose (1,6-DGG *m*/*z* 483), βG and 1,3,6-trigalloylglucose (1,3,6-TGG *m*/*z* 635), and βG and 1,2,3,6-tetragalloylglucose (1,2,3,6-TeGG *m*/*z* 787) as substrates, respectively (left to right). C and D, EIC diagrams of hydrolysis products of recombinant FaCXE proteins purified from *E. coli* using 1,2,3,4,6-PGG (*m*/*z* 939) and casuarictin-1 (*m*/*z* 935) as substrates, respectively. a, Diagrams of recombinant FaCXE proteins hydrolyzing 1,2,3,4,6-PGG and casuarictin-1 to GA and EA, respectively. b, Product analysis of FaCXEs hydrolyzing 1,2,3,4,6-PGG and casuarictin-1, respectively. 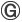 represents a gallic acyl group. The empty vector (EV) denotes a negative control.

A homologous gene (*FaGT2*) of FaU*GT84A22-1* has been reported to catalyze the formation of glucose esters from a variety of (hydroxyl)benzoic and (hydroxyl)cinnamic acids including GA [27]. The FaUGT84A22-1 recombinant protein was expressed in an *Escherichia coli* system, and the *in vitro* enzymatic assays revealed that it can catalyze the glycosylation reaction of GA to synthesize βG, with UDP glucose (UDPG) as a galloyl donor (Fig. 4A).

*FaSCPL2* and *FaSCPL5* are homologous to *CsSCPL4* and *CsSCPL5* from *Camelia sinensis*, jointly involved in the synthesis of galloyl catechins. It has been verified that CsSCPL4 exhibits catalytic acyltransferase activity, whereas CsSCPL5 is a noncatalytic chaperone homologous protein (NCCP) [21]. To assess whether the SCPL-ATs in strawberry plants have similar galloylation functions, the ORF sequences of these genes were successfully constructed into the vector pCAMBIA1305 using recombinant technology and then transformed into *Agrobacterium* GV3101 (psoup-p19). *Agrobacterium* GV3101 carrying pCAMBIA1305-*SCPL-ATs* and pCAMBIA1305 (empty vector, EV) was injected individually or with pCAMBIA1305-*SCPL-ATs* into *Nicotiana benthamiana* leaves for transient gene expression. After three days, *N. benthamiana* leaves were collected to extract crude protein for enzyme activity assay. The results indicated that the co-expressed *FaSCPL2* and *FaSCPL5* catalyzed the galloylation of epicatechins to form epicatechin (ECG) and epigallocatechin gallate (EGCG) using βG and epicatechins as substrates. However, when βG and 1,3,6-tetragalloylglucose (1,3,6-TeGG) or 1,2,3,6-tetragalloylglucose (1,2,3,6-TeGG) were used as substrates, no simple HTs were synthesized (Supplementary Data Fig. S5). This suggests that, similar to tea plants, the functions of genes in the flavan-3-ol galloylation gene cluster in strawberry plants are conserved: FaSCPL2 catalyzes the galloylation of flavanols, and FaSCPL5 acts as an NCCP.

According to the strategy of *FaSCPL* expression mentioned above, three *FaSCPL3* genes were expressed in *N. benthamiana* leaves. Among these, only *FaSCPL3*-*1* demonstrated significant catalytic activity. With βG as an acyl donor and an acceptor, and crude protein extracted from *N. benthamiana* leaves expressing pCAMBIA1305-*FaSCPL3*-*1* as the enzyme source for enzyme activity, two DGG products (DGG1 and DGG2) were detected by UPLC-QqQ-MS/MS (Fig. 4B). The retention time of DGG2 was consistent with that of the 1,6-DGG standard. Subsequently, when βG was used as an acyl donor, and 1,6-DGG, 1,3,6-TGG, and 1,2,3,6-TeGG as acyl acceptors, the corresponding products (1,3,6-TGG, 1,2,3,6-TeGG, and 1,2,3,4,6-PGG) were detected. However, no products were observed in the control system (Fig. 4B). These results suggest that when βG acts as an acyl donor, FaSCPL3-1 catalyzes the continuous galloylation of glucose to form PGG. FaSCPL3-1 acts as an HTS responsible for catalyzing the synthesis of simple GTs.

The same experiment was conducted to verify the functions of FaSCPL3-2 and FaSCPL3-3. Unfortunately, no catalytic activity was detected for these two isoforms (Supplementary Data Fig. S6). Amino acid sequence alignment revealed that FaSCPL3-1 exhibited 61.86% similarity to FaSCPL3-2 and 66.79% similarity to FaSCPL3-3. Sequence alignment revealed that all three proteins contained three key catalytic sites. However, there were differences in the residues at the oxygen hole position (Supplementary Data Fig. S7A). The mutation analysis of amino acid residues showed that the oxygen hole position changed from tyrosine (Y) to phenylalanine (F) (Y189F), which abolished enzyme activity in FaSCPL3-1. However, reverse mutations did not restore enzyme activity in FaSCPL3-2 (F207Y) or FaSCPL3-3 (F182Y) (Supplementary Data Fig. S7A). These results suggest that *FaSCPL3-1* is the primary gene responsible for the synthesis of simple GTs in strawberry plants.

FaCXE1 (FaTA) catalyzed the hydrolysis of galloyl derivatives, whereas FaCXE2 and FaCXE3 catalyzed that of volatile esters [26]. ORFs of 15 *FaCXEs* selected from 35 *FaCXE* members were cloned for enzyme activity assays. Following Dai’s method [22], the hydrolytic activity of recombinant proteins expressed in the prokaryotic system of HTs and volatile esters was evaluated using an *in vitro* enzymatic system (Fig. 4C and Supplementary Data Fig. S8). The results showed that FaCXE1, FaCXE2, FaCXE3, FaCXE5 and FaCXE7 exhibited catalytic activity in hydrolyzing PGG to produce multiple products, including TeGGs, TGGs, DGG, MGGs, and GA (Fig. 4C and Supplementary Data Fig. S7). In contrast, recombinant proteins expressed by *FaCXE8*, *FaCXE9*, *FaCXE10*, *FaCXE12*, *FaCXE14*, *FaCXE26*, *FaCXE31* genes did not hydrolyze PGG but hydrolyzed acetate substrates (Supplementary Data Fig. S8). Among these, FaCXE2 and FaCXE3 catalyzed the above two substrates. Notably, the main synthetic products in the FaSCPL3-1 assay did not coincide with the principal hydrolysis products detected in the FaCXEs assay. Several synthesized products (e.g. 1,2,3,6-TeGG) and hydrolyzed products (such as TGG-1) were detected in various strawberry organs (Fig. 4B and C), indicating that the formation of simple GTs in strawberries results from a combination of the galloylation of FaSCPL3-1 and the degalloylation mediated by FaCXEs.

To assess whether CXEs can hydrolyze ETs and release EA, 1,2,3,4,6-PGG oxidation products with a parent ion of *m*/*z* 935 (identified as casuarictin-1 in strawberries) were used as substrates in the hydrolysis experiments. Two products with a parent ion of *m*/*z* 783 (identified as pedunculagin-1 and pedunculagin-2 in strawberries) were detected, corresponding to the loss of a galloyl group from casuarictin-1 (Fig. 4D). As the reaction progressed, the primary product, pedunculagins, decreased, and its further hydrolysis products, HHDP-glucose with a parent ion of *m*/*z* 481 (formed by the removal of an EA group from pedunculagins) and EA with a parent ion of *m*/*z* 301, gradually increased (Supplementary Data Fig. S9).

The above experimental results were integrated into a G-DG cycle model. GA from the shikimic acid pathway is glycosylated by FaUGT84A22-1 to form βG. FaSCPL3-1 utilizes βG as an acyl donor to catalyze the synthesis of various galloylated compounds, such as 1,6-DGG, 1,3,6-TGG, 1,2,3,6-TeGG, and 1,2,3,4,6-PGG. FaCXE1/FaCXE3/FaCXE7 catalyzes the hydrolysis of 1,2,3,4,6-PGG or other simple GTs and ETs into a series of products, releasing GA and EA. GA from the hydrolysis reaction further forms βG under the action of FaUGT84A22-1, which is recycled into the cycle (Fig. 1).

### Effect of *FvSCPL3*-*1*-RNAi and *FaCXE7*-OE on HT synthesis in strawberry plants

**Figure 5:**
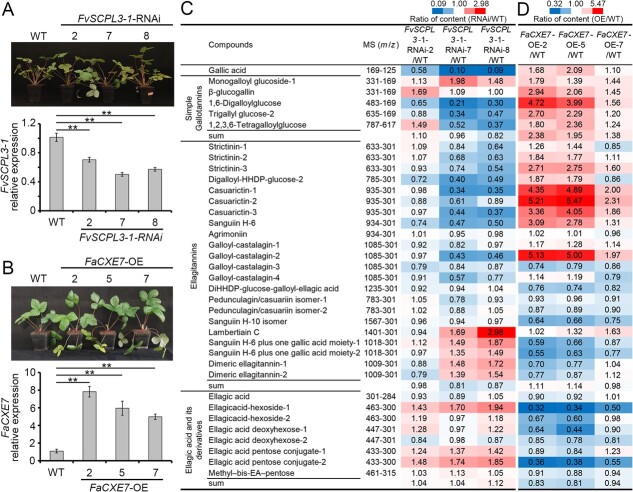
Effect of *FvSCPL3*-*1*-RNAi and *FaCXE7*-OE on the accumulation of HTs in strawberry plants. A, Phenotype of WT and *FvSCPL3*-*1*-RNAi lines and relative expression of *FvSCPL3*-*1* in WT and *FvSCPL3*-*1*-RNAi lines. Data were presented as mean ± SE of three biological replicates. Statistical significance was analyzed using the Student’s *t*-test: ^*^P < 0.05, ^**^P < 0.01. B, Phenotype of WT and *FaCXE7* overexpressing lines and relative expression of *FaCXE7* in WT and *FaCXE7* overexpressing lines. C and D, Content analysis of HTs in *FvSCPL3*-*1*-RNAi and *FaCXE7* overexpressing transgenic lines relative to WT, respectively. The data represent the ratio of peak areas of transgenic strawberry plants and WT.

In order to evaluate the function of FvSCPL3-1 in HT biosynthesis in strawberry plants, *FvSCPL3*-*1*-RNAi transgenic *F. vesca* was generated by transforming strawberry using *Agrobacterium tumefaciens* GV3101 carrying the silencing vector *FvSCPL3*-*1*-pK7GWIWG2(I) (Fig. 5A). A 301-bp fragment from the N-terminal region of *FvSCPL3*-*1* was used to interfere with the target gene (Supplementary Data Fig. S10). The qRT-PCR results revealed that the transcript levels of *FvSCPL3*-*1* in the leaves of *FvSCPL3*-*1*-RNAi transgenic strawberry plants were reduced by 30%–50% compared to wild type (WT) plants. The appearance and growth of *FvSCPL3*-*1*-RNAi transgenic plants did not differ significantly from those of the control plants.

Additionally, to further investigate the role of *CXEs* in HT accumulation in strawberry plants, an *Agrobacterium*-mediated genetic manipulation experiment was performed to obtain transgenic strawberries with overexpression of *FaCXE7*, referred to as *FaCXE7*-OE strawberry transgenic lines (Fig. 5B). qRT-PCR results showed that the transcript levels of *FaCXE7* in the leaves of *FaCXE7*-OE transgenic strawberry plants were 4–8 times higher than those of WT plants. The appearance and growth of *FaCXE7*-OE transgenic plants differed significantly from those of control plants. Morphological observations revealed that the stems of transgenic plants were soft and droopy, and the size and weight of the seeds were increased compared to those of the WT (Supplementary Data Fig. S11).

HT compounds extracted from the leaves of *FvSCPL3*-*1*-RNAi and *FaCXE7*-OE transgenic plants and WT plants were quantitatively detected using Q-TOF-UPLC/MS (Fig. 5C and D; Supplementary Data Tables S6 and S7). The results showed that, compared with the control, the content of HT compounds in the leaves of *FvSCPL3*-*1*-RNAi and *FaCXE7*-OE transgenic plants exhibited opposite trends. For instance, the content of simple GT compounds, such as GA, 1,6-DGG, TGG-2, and 1,2,3,6-TeGG, in the leaves of *FvSCPL3*-*1*-RNAi transgenic plants was significantly lower than that in WT plants, whereas their content in *FaCXE7*-OE transgenic plants was higher than in the control plants. In both transgenic plants, the levels of MGG-1 and βG in the GA group were higher than those in the control plants. Both MGG-1 and βG are likely synthesized from GA through glycosylation catalyzed by plant UGTs, which may also explain the very low content of the hydrolysis product, GA, in *FvSCPL3*-*1*-RNAi transgenic plants.

Except for ET compounds with high molecular weights (such as those with parent ions *m*/*z* 1401.1265, *m*/*z* 1009.0846, and *m*/*z* 1018.089x), the changes in the content of ET compounds in the leaves of *FaCXE7*-OE and *FvSCPL3*-*1*-RNAi transgenic plants were similar to those of simple GTs. This suggests that both FaCXE7 and FaSCPL3-1 not only regulate the biosynthesis of simple GT compounds but also control the accumulation of oxidation products (monomeric ETs) of simple GT compounds.

Although EA is produced from the hydrolysis of ETs, metabolic data revealed that the changes in glycoside compound content were not consistent with the changes in ETs and GTs in the two transgenic lines. The content of EA derivatives, such as ellagic acid hexoses, ellagic acid deoxyhexoses, and ellagic acid pentose conjugate-2, in the leaves of *FaCXE7*-OE transgenic strawberry plants was significantly lower than that in the control plants. In contrast, the content of these compounds in *FvSCPL3*-*1*-RNAi transgenic plants was significantly higher than that in the control plants. These observations suggest that FaSCPL3-1 and FaCXE7 may regulate the accumulation of EA glycosides.

In summary, genetic manipulation of *FvSCPL3*-*1*-RNAi and *FaCXE7*-OE had opposite effects on the accumulation of HTs and EA derivatives in transgenic plants. Both genes positively regulate the synthesis of simple GTs and monomeric ET compounds. These findings also partially validated our previous results based on transient overexpression and silencing of *CXEs* in strawberry plants, tea plants, and bayberry plants [22, 23]. It is speculated that the hydrolysis of FaCXE7 increases the concentration of the acyl donor βG and accelerates the G-DG cycle.

### *FaCXE7-OE* causes crosstalk with immediate metabolic neighborhoods (such as the lignin pathway in strawberry plants)

Interestingly, the overexpression of *FaCXE7* significantly promoted the growth and development of transgenic strawberries (Fig. 5B). In particular, the phenomenon of soft and droopy stems raises questions about whether lignin synthesis is affected. To exclude the lignin pathway as a direct target of *FaCXE7*-OE, *FaCXE7* was overexpressed in *Arabidopsis* under the action of the 35S promoter (Supplementary Data Fig. S12). The results showed no significant difference in the appearance or lignin content of transgenic plants compared to the control. These results demonstrate that the lignin pathway in plants is not a direct target of FaCXE7.

**Figure 6:**
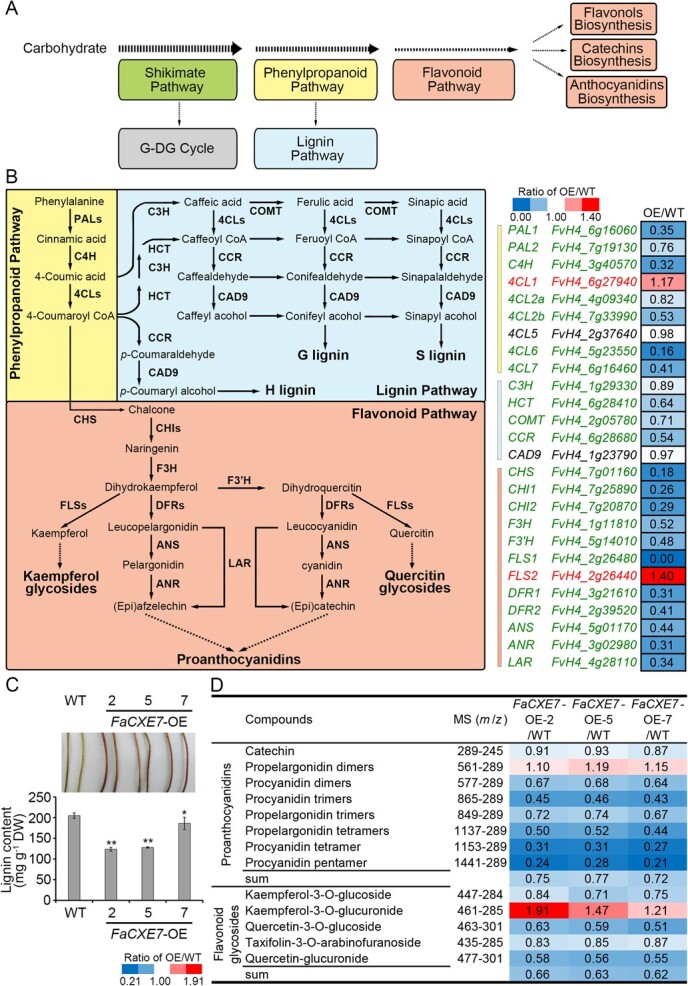
FaCXE7 causes crosstalk with other metabolic pathways in strawberry plants. A, Schematic diagram of the G-DG cycle and its immediate metabolic neighborhoods in strawberry plants. B, Metabolic pathways of phenylpropanoid, lignin, and flavonoid and expression patterns of critical genes (left). The heatmap reflects the *FaCXE7*-OE/WT expression (FPKM values) ratio based on the transcriptome data from the three pathways (right). C, Changes in the contents of lignin, PAs, and flavonoid metabolites. Data were presented as mean ± SE of three biological replicates. Statistical significance was analyzed using Student’s *t*-test: ^*^P < 0.05, ^**^P < 0.01. PAL, phenylalanine ammonia lyase; C4H, cinnamate 4-hydroxylase; 4CL, 4-coumaroyl-CoA ligase; C3H, *p*-coumaroyl-CoA 3-hydroxylase; HCT, hydroxycinnamoyl-CoA shikimate/quinate hydroxycinnamoyltranferase; COMT, caffeic acid O-methyltransferase; CCR, cinnamoyl CoA reductase; CAD9, cinnamyl alcohol dehydrogenase 9; CHS, chalcone synthase; CHI, chalcone isomerase; F3H, flavanone 3-hydroxylase; F3’H, flavonoid 3′-hydroxylase; FLS, flavonol synthase; DFR, dihydroflavonol reductase; ANS, anthocyanidin synthase; ANR, anthocyanidin reductase; LAR, leucoanthocyanidin reductase.

It is hypothesized that *FaCXE7* overexpression can promote the flow rate of the G-DG cycle, thereby readjusting the distribution of glycogen in plants and affecting the immediate metabolic neighborhoods of the G-DG cycle. Such changes may influence plant growth and development. To confirm whether *FaCXE7*-OE causes crosstalk with the immediate metabolic neighborhoods of the G-DG cycle (Fig. 6A), the gene expression levels and compound content of these neighborhoods were compared between *FaCXE7*-OE transgenic and control plants (Fig. 6B-D).

Q-TOF-UPLC/MS system was used to analyze leaf extracts, comparing the compound content between *FaCXE7*-OE transgenic plants and the WT plants. The results showed that the levels of lignin, proanthocyanidins (PAs), and flavonoid glycosides in the leaves of *FaCXE7*-OE transgenic strawberry plants were significantly lower than those in the control plants (Fig. 6C and D).

Transcriptome sequencing was used to compare gene expression between *FaCXE7*-OE transgenic and control plants. PCA results based on transcriptomic data presented that *FaCXE7*-OE transgenic plants differed significantly from the control plants, indicating notable differences in their gene expression profiles (Supplementary Data Fig. S13A). Differentially expressed genes (DEGs) were selected based on a false discovery rate (FDR) < 0.05, | log2(fold change) | > 1. Compared with the control, *FaCXE7*-OE plants exhibited upregulation of 756 genes and downregulation of 680 genes (Supplementary Data Fig. S13B). Gene ontology (GO) enrichment analysis of DEGs showed significant enrichment in molecular function. Among the top 20 GO terms, 17 were related to molecular function, and three were linked to cellular components. The most enriched GO terms in molecular function included hydrolase activity (GO: 0004553; GO: 0016798), protein kinase activity (GO: 0004672), transporter activity (GO: 0005215) and catalytic activity (GO: 0003824) (Supplementary Data Fig. S13C and D). These results suggest that these DEGs are primarily involved in hydrolase activity, particularly acting on glycosyl bonds, which contribute to the phenotypes in strawberry plants. KEGG analysis revealed that the DEGs were mainly enriched in secondary metabolic pathways (ko01110) and metabolic pathways (ko01100), including the phenylpropanoid pathway (ko00940), flavonoid pathway (ko00941), starch and sucrose metabolism (ko00500), sesquiterpenoid and triterpenoid biosynthesis (ko00909), and cyanoamino acid metabolism (ko00460) (Supplementary Data Fig. S13E and F). KEGG analysis implies that the metabolic pathways in *FaCXE7*-OE plants are significantly altered compared to those in the WT. *FaCXE7* affected related metabolic processes through the phenylpropanoid and flavonoid pathways in strawberry plants (Fig. 6B-D).

Particularly, in the phenylpropanoid pathway (ko00940), the expression levels of several key genes, including phenylalanine ammonia-lyase (*PAL*), cinnamate 4-hydroxylase (*C4H*), 4-coumaroyl-CoA ligase (*4CL*), *p*-coumaroyl-CoA 3-hydroxylase (*C3H*), hydroxycinnamoyl-CoA shikimate/quinate hydroxycinnamoyltranferase (*HCT*), caffeic acid O-methyltransferase (*COMT*), cinnamoyl CoA reductase (*CCR*), and cinnamyl alcohol dehydrogenase 9 (*CAD9*) were lower in *FaCXE7*-OE plants than in the control plants (Fig. 6B), indicating that lignin synthesis was significantly affected in the transgenic plants. This is consistent with the observed reduction in lignin content in *FaCXE7*-OE strawberry plants (Fig. 6C).

Additionally, in transgenic plants, the expression levels of chalcone synthase (*CHS*), chalcone isomerase (*CHI*), flavanone 3-hydroxylase (*F3H*), flavonoid 3*′*-hydroxylase (*F3'H*), flavonol synthase (*FLS*), dihydroflavonol 4-reductase (*DFR*), anthocyanin synthase (*ANS*), anthocyanin reductase (*ANR*), and leucoanthocyanin reductase (*LAR*) showed a downward trend in the flavonoid pathway (ko00941) (Fig. 6B). This indicates a significant reduction in flavonoid synthesis, including PAs in the transgenic plants. This correlates with the observed decrease in PAs and flavonoid content (Fig. 6D).

The above transcriptional and metabolism analyses suggest that when overexpression of *FaCXE7* upregulates the G-DG cycle in plants, which in turn downregulates key genes in immediate metabolic neighborhoods, such as lignin and flavonoid metabolism pathways. This effect is likely due to competition for carbon sources between the G-DG cycle and these metabolic pathways, ultimately impacting plant growth and development.

## Discussion

Significant progress has been made in understanding the galloylation and degalloylation mechanisms of galloylated flavan-3-ols in *Camellia sinensis* [19–23]. Through enzyme activity assays and transgenic approaches, FaSCPL3-1 has been confirmed as an HTS responsible for catalyzing the continuous galloylation of simple GTs (Fig. 4B).

As early as the 1980s and 1990s, Gross *et al*. employed protein purification techniques and *in vitro* enzyme assays to investigate the properties of β-glucogallin-dependent galloyltransferases involved in the synthesis of simple GTs in oak leaves and staghorn sumac [18]. The galloylation of hydroxyl groups at various positions on the glucose structure occurs in a position-specific order: 1-OH, 6-OH, 2-OH, 3-OH, and 4-OH. Four high-molecular-weight galloyl transferases were identified, including β-glucogallin: β-glucogallin 6-*O*-galloyltransferase (M*_r_* 400 kDa) [28], β-glucogallin: 1,6-di-*O*-galloylglucose 2-*O*-galloyltransferase (M*_r_* 750 kDa) [29], β-glucogallin: 1,2,6-tri-*O*-galloyl-β-D-glucose 3-*O*-galloyltransferase (M*_r_* 380 kDa) [30], and β-glucogallin: 1,2,3,6-tetra-*O*-galloylglucose 4-*O*-galloyltransferase (M*_r_* 260 kDa) [31]. From the characteristics of the molecular weight of the enzymes, it is apparent that these four enzyme proteins were not derived from the same gene. However, in contrast, the protein cloned from the *FaSCPL3*-*1* gene in strawberry plants catalyzed the synthesis of PGG from DGG, facilitating a continuous four-step reaction in the biosynthesis of simple GTs (Fig. 4B). In our previous study, the purified prokaryote-expressed SCPL-ATs did not exhibit acyltransferase activity. Therefore, this study employed transient overexpression in *N. benthamiana* leaves to obtain crude protein extracts for further analysis. However, due to difficulties in obtaining purified proteins, the enzymatic kinetic characteristics of FaSCPL3-1 could not be determined, and its optimal natural substrate remains obscure. A comparison of relative peak values of the reaction products disclosed that 1,2,3,6-TeGG and 1,3,6-TGG were the predominant enzymatic products (Fig. 4B). Currently, studies on FaSCPL3-1 are limited due to challenges in protein purification. Further work will focus on improving methods for isolating FaSCPL3-1 proteins and identifying additional galloyltransferase genes involved in catalyzing HT synthesis.

Moreover, this study validated that the expression of *FaSCPL2* requires the cooperation of *FaSCPL5* for the synthesis of galloylated flavan-3-ols (Supplementary Data Fig. S4). This agrees with a previous report [21]. However, it remains unclear why *FaSCPL3*-*1*, which is responsible for HT synthesis, does not require the involvement of *FaSCPL5* (Fig. 4B). The function of the SCPL5 homologous gene appears to be conserved across various plant species, such as tea, strawberries, and *C. oleifera*, where it acts as a noncatalytic companion paralogs associated with flavan-3-ol galloylation (NCCP) [21]. This study demonstrated that the SCPL5 homologous genes evolved from non-functionalized genes within a flavan-3-ol galloylation-related SCPL gene cluster, which exists in several plants. In contrast, the SCPL3 homologous genes do not belong to this gene cluster [32]. This flavan-3-ol galloylation-related gene cluster, including *UGT84A22* and *SCPL-AT* orthologs, is adjacent to chromosomes in tannin-rich plants, such as tea plants, *Vitis vinifera*, and *Punica granatum* [32].

CXE is a member of the hydrolytic enzyme superfamily, known for its diverse physiological functions in plants [33–36]. Members of the plant CXE family can be classified into seven subgroups, with the third subgroup containing the highest number of CXE members [26, 37–41]. Plant TA, belonging to the third subgroup of the CXE family, can hydrolyze ester bonds in both HTs and galloylated flavan-3-ols structures, facilitating the removal of GA [22, 23]. This study found that multiple recombinant proteins cloned from the *FaCXE* genes exhibited TA activity. Specifically, FaCXE1/FaCXE3/FaCXE7, all members of the third subgroup, catalyzed the hydrolysis or degalloylation of simple GTs and ETs, respectively (Fig. 4C). These genes are located in a gene cluster composed of ten third subgroup *FaCXEs* on chromosome 2 of strawberry (Fig. 3C). This study observed that the *FaCXEs* in this gene cluster underwent functional mutations. FaCXE1 (or FaTA) has been reported to be involved in the hydrolysis of galloylated flavan-3-ols and HTs [22], whereas FaCXE2 and FaCXE3 are implicated in the hydrolysis of ethyl ester aroma compounds [26]. This study identified that FaCXE8, FaCXE9, FaCXE10, FaCXE12, FaCXE14, FaCXE26, and FaCXE31 can hydrolyze ethyl esters (Supplementary Data Fig. S8). Similarly, other studies have shown that the third subgroup of CXE in plants exhibits substrate diversity and has multiple effects on plant physiological activities. TA genes involved in HT hydrolysis have been found in tannin-rich plant species [22]. CXEs also play a role in the hydrolysis of volatile acetate esters in some fruit species, such as strawberries, apples, tomatoes, and peaches [26, 39, 42–44]. Furthermore, some CXEs in the third subgroup lacked CXE activity but function as 2-hydroxyisoflavanone dehydratases [45, 46].

Based on *in vitro* enzymatic assays and transient overexpression and interference gene expression assays [22, 23], it is speculated that there is a G-DG cycle of galloyl compounds in tannin-rich plants (Fig. 1). CsTA, a hydrolase with promiscuous acyltransferase activity, can accelerate the utilization of GA in this cycle, promoting the accumulation of ETs in strawberries and GTs and flavan-3-ol gallates in the leaves of tea and bayberry plants [23]. The functions of FaSCPL3-1 and FaCXE7 in the synthesis and metabolism of HTs in strawberries were explored using the stable genetic transformation system in strawberries (Fig. 5). Quantitative analysis of metabolites revealed that, compared to the control plants, the content of simple GT compounds and some ETs, such as strictinins, digalloyl-HHDP-glucose-2, and casuarictins, decreased in *FvSCPL3*-RNAi transgenic plants and increased in *FaCXE7*-OE transgenic strawberries (Fig. 5C and D). This suggests although FaCXE7 is a hydrolase, its increased expression promotes the accumulation of simple GTs and ETs in strawberry plants. This finding is consistent with our previous conclusion [22].

This study highlights the profound impact of *FaCXE*7 gene expression on the growth and development of strawberry plants. A key question is how *FaCXE7* influences plant growth and development. Transcriptome sequencing analysis revealed that the expression of several genes in the lignin synthesis pathway was significantly downregulated in the stems of *FaCXE7*-OE transgenic plants (Fig. 6B). For the first time, this study discovered that FaCXE7, as a plant TA, not only promotes HT synthesis but also interferes with plant growth and development by reducing lignin synthesis. It is hypothesized that overexpression of *FaCXE7* diverts carbohydrate flux away from the lignin pathway rather than directly inhibiting lignin substrate synthesis. When *FaCXE7* was overexpressed in *Arabidopsis*, a species lacking the HT synthesis pathway, transgenic plants exhibited no abnormal growth or development symptoms (Supplementary Data Fig. S12).

In summary, HTs affect the favor of strawberry fruits, provide health benefits for consumers, and contribute to plant disease resistance. This study verified that FaUGT84A22, FaSCPL-ATs, and CXEs constitute a G-DG cycle that regulates the biosynthesis of HTs in strawberry plants. Additionally, overexpression of *FaCXE7* interferes with plant growth and development by downregulating the lignin biosynthesis pathway. These findings advance the understanding of HT accumulation in strawberry plants. Furthermore, the G-DG cycle can be leveraged to enhance strawberry quality by optimizing flavor and boosting disease resistance through targeted genetic modifications, thereby providing valuable molecular insights into the improvement of strawberry fruit quality.

## Materials and methods

### Plant materials and growth conditions

Octoploid strawberry (*Fragaria* × *ananassa* Duch.) Benihoppe and “Alpine” strawberry (*Fragaria vesca*) plants were cultivated in a greenhouse at 21 ± 2°C under a 16-h/8-h light/dark photoperiod. Various tissues from Benihoppe, including leaves, petioles, flowers, small green fruits (SG, nine-day after flowering), white fruits (white, 24-day after flowering), red fruits (red, 30-day after flowering), and roots, were collected, frozen in liquid nitrogen, and then stored at −80°C until further use.

*N. benthamiana* and *Arabidopsis thaliana* plants were grown in a greenhouse under a photoperiod of 16-h light and 8-h darkness at 23 ± 2°C.

### Chemicals and standards

HPLC-grade methanol, acetonitrile, and acetic acid were purchased from Tedia (Fairfield, Ohio, USA).

(−)-Epicatechin (EC, CAS No: 490-46-0), (−)-epicatechin gallate (ECG, CAS No: 1257-08-5), (−)-epigallocatechin (EGC, CAS No: 970-74-1), (−)-epigallocatechin gallate (EGCG, CAS No: 989-51-5) were obtained from Sigma-Aldrich (St. Louis, MO, USA). 1,2,3,4,6-*O*-pentagalloylglucose (PGG, CAS No: 14937-32-7) standard came from Shanghai Yuanye Bio-Technology Co., Ltd. (Shanghai, China). 1,3,6-*O*-trigalloylglucose (TGG, CAS No: 18483-17-5) and 1,2,3,6-*O*-tetragalloylglucose (TeGG, CAS No: 79886-50-3) standards were purchased from ChemFaces (Wuhan, China). Casuarictin (CAS No: 79786-00-8) was obtained from Chengdu Bio-Technology Co. Ltd. (Chengdu, China). βG (CAS No: 13405-60-2) and 1,6-*O*-digalloylglucoses (1,6-DGG, CAS No: 23363-08-8) were synthesized in the laboratory.

### Extraction and identification of phenolic compounds from strawberry tissues

Phenolic compounds were extracted from strawberry tissues, including leaves, petioles, flowers, small green fruits, white fruits, red fruits, and roots, using a previously described method with minor modifications. Specifically, 0.1 g of freeze-dried strawberry powder was suspended in 700 μl of 70% acetone. Then, this mixture was placed in a 2.0 ml polypropylene tube containing zirconium beads and vortexed at 20 Hz for 10 min. After centrifuged at 14 975 g for 5 min, the supernatant was transferred to a new tube. The residue was extracted twice. All extracts were combined to achieve a final volume of 1.0 ml and were centrifuged at 14 975 g for 15 min. The clear supernatant was used for Q-TOF-UPLC/MS analysis.

The phenolic compounds in the extracts were quantitatively analyzed using an Agilent 6545 Q-TOF-UPLC/MS system (Agilent Technologies, Palo Alto, CA, USA). The system adopted an Agilent 20RBAX RRHD Eclipse Plus C18 column (1.8 μm, 100 × 2.1 mm) with a flow rate of 0.2 ml min^−1^ at 40°C. The mobile phase consisted of 0.4% (v/v) acetic acid (A) and 100% acetonitrile (B). The elution gradient was as follows: 5%–10% B for 0–10 min, 10%–12.5% B for 10–22 min, 12.5%–30% B for 22–42 min, 30%–60% B for 42–45 min, 60%–5% B for 45–57 min and maintained 5% B for 3 min. The injection volume was 3 μl. The Mass acquisition was performed in negative ionization mode. The other parameters included dry gas flow rate: 8 l min^−1^; dry gas temperature: 325°C; nebulizer pressure: 45 psi; capillary voltage: 3500 V. The mass range was set at m/z 100–1700. Phenolic compounds were analyzed and quantified using corresponding standards and characteristic ion fragments.

### Gene sequence analysis

Strawberry *SCPL-AT* genes were identified from the strawberry genome available at Genome Database for Rosaceae (https://www.rosaceae.org/) using tea (*C. sinensis*) CsSCPL4 and CsSCPL5 as query sequences. Tea plant *SCPL-AT* genes were screened in the tea plant genome database (http://eplant.njau.edu.cn/tea/index.html). SCPL-AT member from Grape (*V. vinifera*), pomegranate (*P. granatum*), eucalypts (**E*. grandis*), oaks (*Q. suber*) and walnuts (**J*. regia*) were retrieved from the National Center for Biotechnology Information database (https://www.ncbi.nlm.nih.gov/genome/?term=).

From the peach genome database at Phytozome and Tair, CXE genes were identified from the strawberry genome through BLAST analysis using the protein sequences of PpCXE1 and AtCXEs as queries.

Phylogenetic analysis was performed using the neighbor-joining method with MEGA 5.0, employing 1000 bootstrap replicates. The accession numbers of *UGT84A22*, *SCPL-AT*, and *CXE* genes of strawberry, tea, grape, pomegranate, eucalypts, oaks, and walnuts species are detailed in Supplementary Data Table S3a. Chromosome distributions of *SCPL-AT, UGT84A22*, and *CXE* were visualized using Genelibs.

### Gene cloning, RNA-seq, and data analysis

Total RNA was extracted using the FastPure® Universal Plant Total RNA Isolation Kit (Vazyme) following the manufacturer’s instructions. First-strand cDNA was synthesized from 500 ng RNA using the PrimeScript RT Reagent Kit (Takara, Japan). The ORFs of candidate genes (*UGT84A22*, *SCPL-AT*, and *CXE*) in the Benihoppe strawberry cultivar were amplified using Phusion high-fidelity polymerase (Thermo Scientific), complied with the manufacturer’s protocol. Specific primers for gene cloning are listed in Supplementary Data Table S5.

RNA sequencing was conducted using the Illumina HiseqTM 2500/4000 (Gene Denovo Biotechnology Co., Ltd. Guangzhou, China). Paired-end clean reads were mapped to the diploid strawberry *F. vesca* reference genome (https://www.rosaceae.org/species/fragaria_vesca/genome_v4.0.a2) using HISAT2. 2.4 [47]. Three biological replicates of various strawberry tissue (leaves, petioles, flowers, small green fruits, white fruits, red fruits, and roots), transgenic, and WT plants were subjected to RNA-seq.

Expression patterns of *UGT*, *SCPL-AT,* and *CXE* genes in strawberry tissues were analyzed using FPKM values derived from the RNA-seq data. A heatmap for correlation analysis was generated using TBtools software [48]. The correlation between the relative peak areas of ETs and the expression levels of *UGT*, *SCPL-AT*, and *CXE* genes was assessed using SIMCA 14.0.

### Transient expression of SCPL proteins in *N. benthamiana*

Through recombinant technology, the ORFs of *FaSCPL2*, *FaSCPL5*, and *FaSCPL3s* were cloned into the pCAMBIA1305 vector, driven by the CaMV 35S promoter at the XbaI and BamHI sites. The resulting fusion vectors were individually transformed into *A. tumefaciens* strain GV3101 (pSoup-p19) (Shanghai Weidi Biotechnology, China). Positive *Agrobacterium* GV3101 cultures were suspended in injection buffer (10 mM MES, 10 mM MgCl_2_, and 100 μM acetosyringone; pH 5.6) to reach an OD_600_ of approximately 0.8. The transient expression and protein extraction protocols for SCPL proteins in *N. benthamiana* were performed as previously described [21]. The primer pairs used for vector construction are listed in Supplementary Data Table S5.

### Prokaryotic expression and purification of CXE proteins

The ORFs of *FaCXEs* were cloned into the pRSF-Duet expression vector (Novagen, Schwalbach, Germany) with 6x N-terminal His-tags at BamHI and PstI sites using recombinant technology. The recombinant vectors were then transferred into *E. coli* BL21 (DE3) (Transgen, Beijing, China). Positive transformed cells were cultured overnight at 37°C in Luria–Bertani liquid medium containing 50 μg ml^−1^ kanamycin. Subsequently, the cultures were diluted and incubated at 37°C, shaking at 200 rpm until the OD_600_ reached 0.6–0.8. After adding 0.1 mM isopropyl-β-D-thiogalactopyranoside, the cultures were further incubated overnight at 16°C to induce protein expression. The fusion proteins were purified using His-tag affinity chromatography following the manufacturer’s instructions. Protein concentration and size were determined using a photometric method [49] and confirmed by sodium dodecyl sulfate-polyacrylamide gel electrophoresis.

### Enzymatic activity assays

The enzyme activities of FaSCPL2 and FaSCPL5 were measured according to Yao et al. [21] with minor modifications. Briefly, each 100 μl reaction mixtures contained 50 mM phosphate buffer (pH 6.0), 0.4 mM EC or EGC, 0.4 mM βG, 2 mM ascorbic acid, and 60 μg of desalted protein. Then, the reaction mixture was incubated at 40°C for 3 h and then terminated by adding an equal volume of methanol.

For the enzyme assays of FaSCPL3s, a 100 μl reaction mixture was prepared containing 50 mM phosphate buffer (pH 6.0), with 0.4 mM βG as galloyl donor and 0.4 mM 1,6-DGG, or 1,3,6-TGG, or 1,2,3,6-TeGG as galloyl acceptor. Additionally, 2 mM ascorbic acid and 60 μg of desalted protein were added. The mixture was then incubated at 40°C for 1 h. Then, the reaction was stopped by adding an equal volume of methanol.

For the hydrolysis reactions of FaCXEs, each 50 μl of reaction mixture contained 200 μM of either 1,2,3,4,6-PGG or casuarictin-1 as the substrate, 1 mM ascorbic acid, and 0.5–30 μg of purified recombinant FaCXEs in 50 mM phosphate buffer (pH 7.4). The reaction mixtures were incubated at 35°C for 10 min and terminated by the addition of methanol.

Boiled proteins were employed as the controls. All reactions were centrifuged at 14 975 g at 20°C for 15 min. The supernatant was analyzed using UPLC UltiMate 3000 and UPLC-QqQ-MS/MS 6460, as previously reported [21]. Enzyme reaction products were separated using reverse-phase UPLC on an Agilent 20RBAX RRHD Eclipse Plus C18 column (2.7 μm, 200 × 4.6 mm) at a flow rate of 0.4 mL min^−1^ and a column temperature of 40°C. The elution gradient was as follows: 1%–10% B for 0–10 min, 10%–35% B for 5–20 min, 35%–10% B for 20–21 min, and 1% B for 2 min. The injection volume was 5 μl. Solvent A adopted in UPLC and UPLC-QqQ-MS/MS was 1% or 0.4% (v/v) acetic acid, respectively, and solvent B for both was 100% acetonitrile.

### Site-directed mutation of *FaSCPL3s*

Based on protein sequence analysis of FaSCPL3s, gene-specific primers were designed to introduce mutations at the oxyanion-binding site. Specific mutations were introduced into the *FaSCPL3*-*1* (Y189F), *FaSCPL3*-*2* (F207Y), and *FaSCPL3*-*3* (F182Y) genes using overlap extension PCR. After sequence validation, the mutated cDNAs were cloned into the pCAMBIA1305 vector for recombinant protein expression in *N. benthamiana*. Gene-specific mutation primers are detailed in Supplementary Data Table S5.

### Vector construction and stable transgene in strawberry plants

The silencing and overexpression vectors were constructed using pK7GWIWG2(I) and pCAMBIA2300 vectors, respectively.

#### Gene silencing

The conserved 301 bp region of *FvSCPL3*-*1* was amplified using specific primers (Table S5). The *FvSCPL3*-*1* fragment with an attB linker was then inserted into the entry vector pDNOR207 using the Gateway BP reaction kit (Invitrogen). After sequence confirmation, the binary vector pK7GWIWG2(I) was used to construct the *FvSCPL3*-*1*-pK7GWIWG2(I) vector using the Gateway LR reaction kit (Invitrogen). The RNAi construct was transformed into *Agrobacterium* GV3101 competent cells.

#### Gene overexpressing

Through recombinant technology, the ORF sequence of *FaCXE7* was inserted into the overexpression vector pCAMBIA2300 at XbaI and PstI sites. The *FaCXE7*-pCAMBIA2300 vector was transformed into *Agrobacterium* GV3101 competent cells (Shanghai Weidi Biotechnology, China).

The diploid strawberry “Alpine” was transformed using positive GV3101 colonies harboring *FvSCPL3*-*1*-pK7GWIWG2(I) and *FaCXE7*-pCAMBIA2300. *A. tumefaciens*-mediated transformation of strawberry was performed as previously reported [50]. Briefly, leaf slices of “Alpine” were co-cultivated with GV3101 (OD_600_ = 0.3) in Murashige and Skoog (MS) medium containing 2 mg/L TDZ, 0.2 mg/L IBA, 2% sucrose, and 0.7% agar for 3 days. Then, the leaf explants were washed with sterilized water and plated on the same MS medium supplemented with 300 mg/L carbenicillin. The explants were transferred to a fresh MS medium comprising 300 mg/L carbenicillin and selected with 25 mg/L kanamycin. The resulting transgenic plants were confirmed by PCR.

### Statistical analysis

Statistical analysis was carried out using SPSS software (version 19) with one-way ANOVA analysis. The data were represented as the mean ± SD. Multiple comparisons were performed using Tukey’s test. Significant differences were represented by different letters at *P* < 0.05. Student’s *t*-test was used for pairwise comparisons, with significance denoted by asterisks: ^*^P < 0.05 and ^**^P < 0.01.

## Supplementary Material

Web_Material_uhae350

## Data Availability

The raw sequencing data from this study have been deposited in the Genome Sequence Archive in BIG Data Center (https://bigd.big.ac.cn/), Beijing Institute of Genomics (BIG), Chinese Academy of Sciences, under the accession number CRA017076.
