# Supplementary material for: Key genes in a “Galloylation-Degalloylation cycle” controlling the synthesis of hydrolyzable tannins in strawberry plants
Source: Hortic Res. 2024 Dec 16;12(4):uhae350. doi: 10.1093/hr/uhae350 (PMC11879120; doi:10.1093/hr/uhae350)
Supplement: Web_Material_uhae350 [file web_material_uhae350.zip › Supplemental Figures.docx]

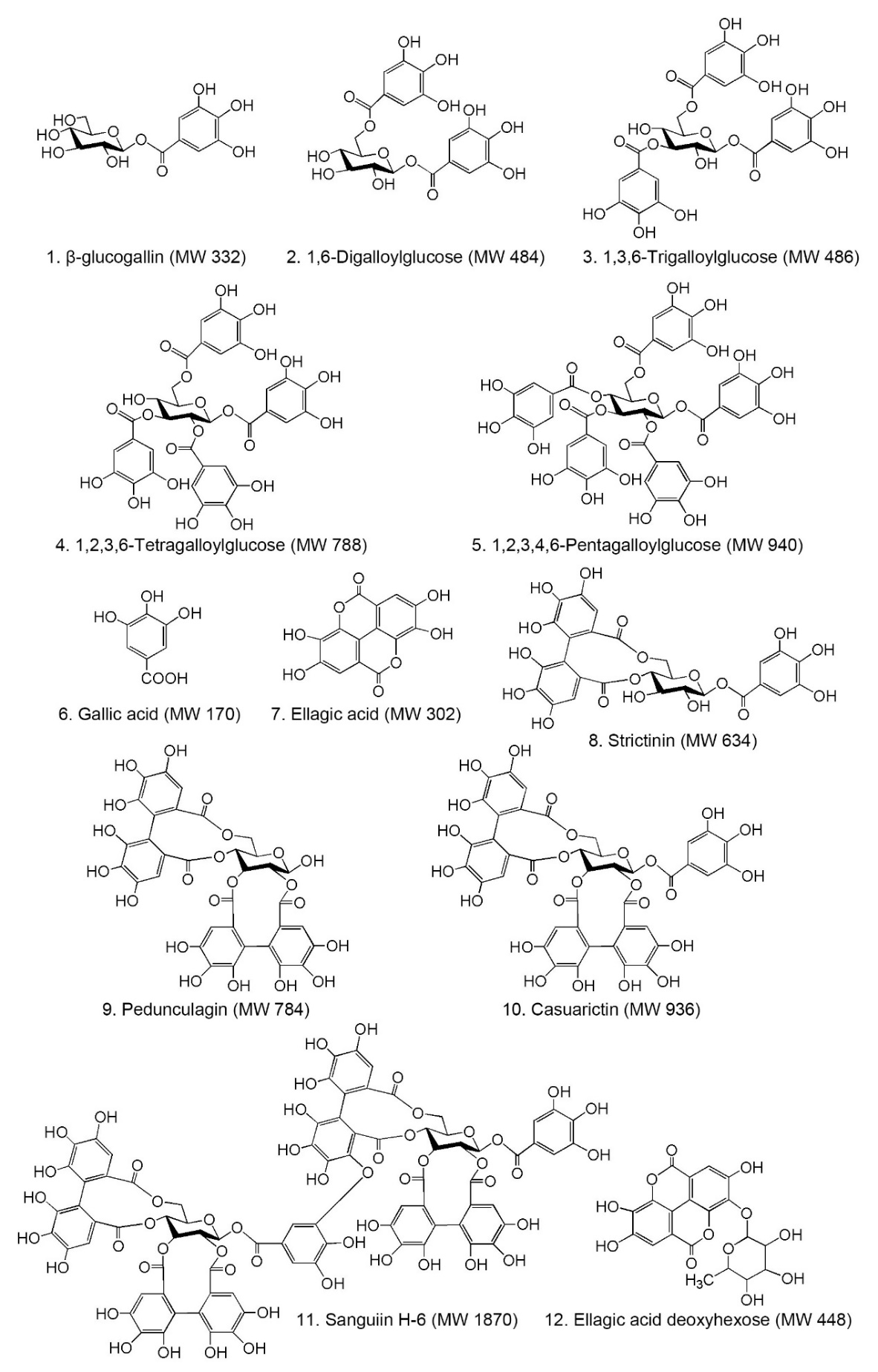
**Key genes in a “Galloylation-Degalloylation Cycle” controlling the synthesis of hydrolyzable tannins in strawberry plants**

**Fig. S1 Schematic structure of hydrolyzable tannins (HTs)**


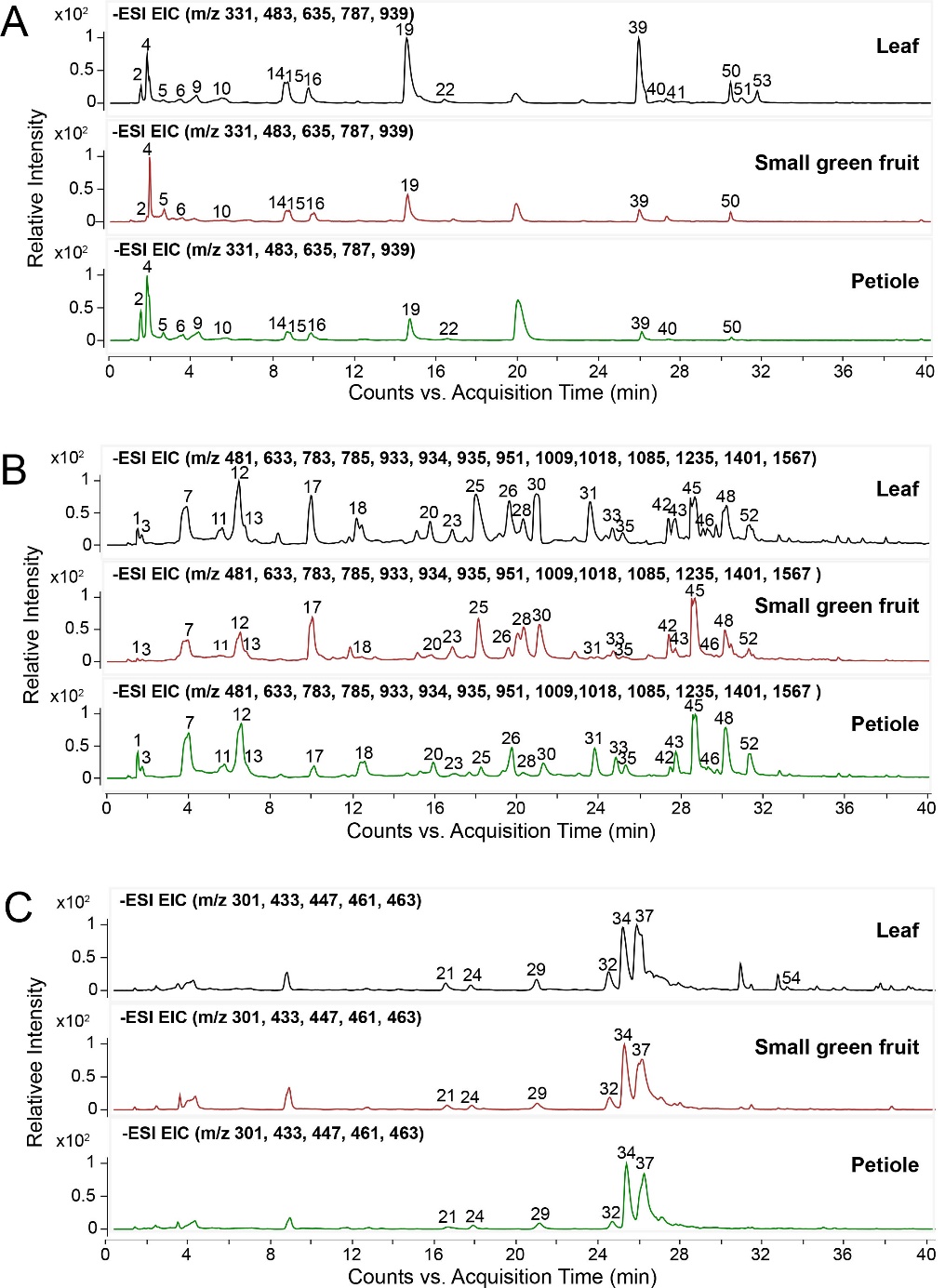


**Fig. S2 EIC diagrams of HTs using Q-TOF-UPLC/MS**

A, EIC diagram of GTs. B, EIC diagram of ETs. C, EIC diagram of EA and their derivatives. Black, red, and green lines indicate the EIC diagrams of compounds from leaves, small green fruits, and petioles, respectively.


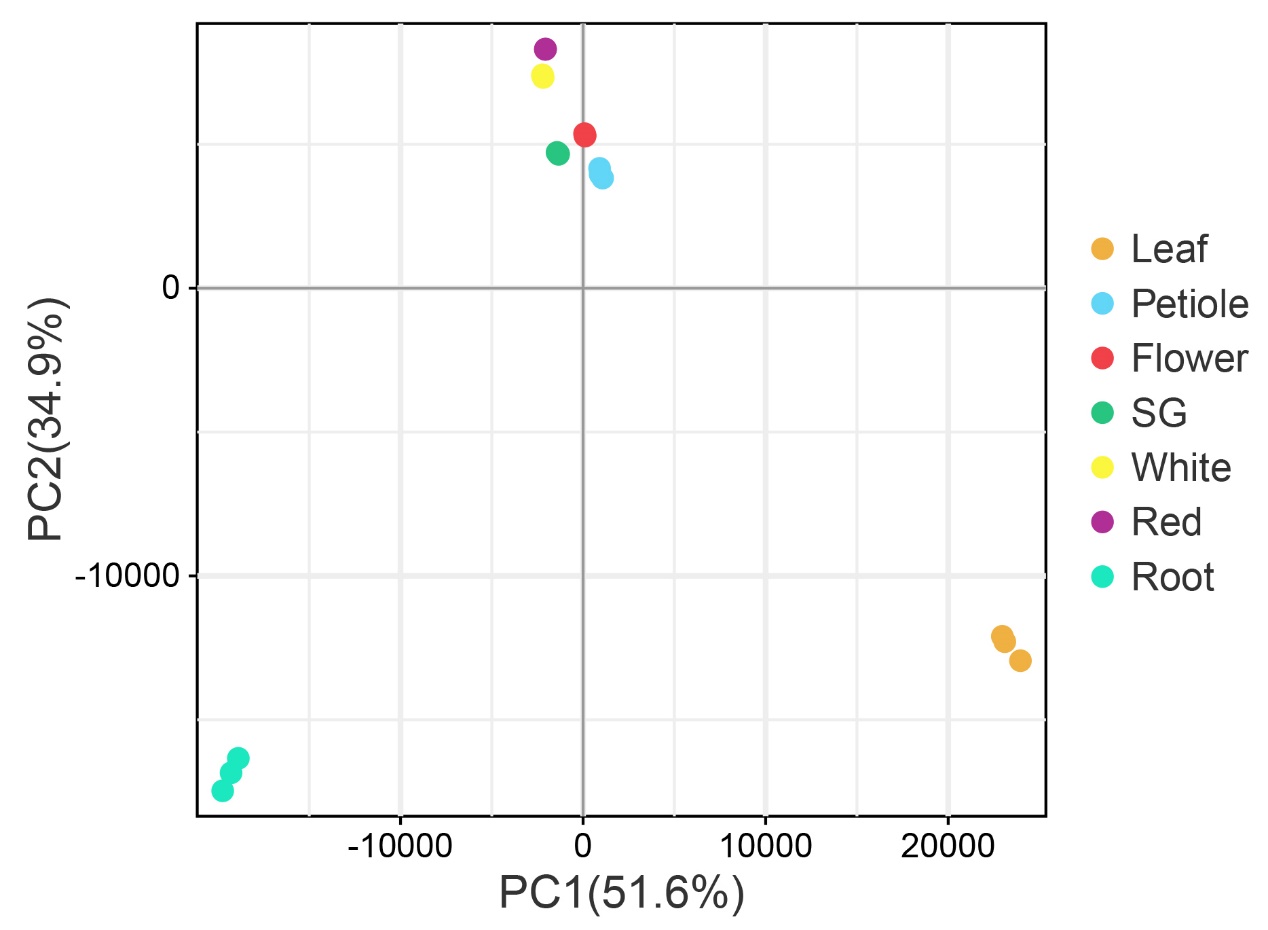


**Fig. S3 Principal Component Analysis (PCA) of different strawberry tissues based on RNA-seq data**

~~
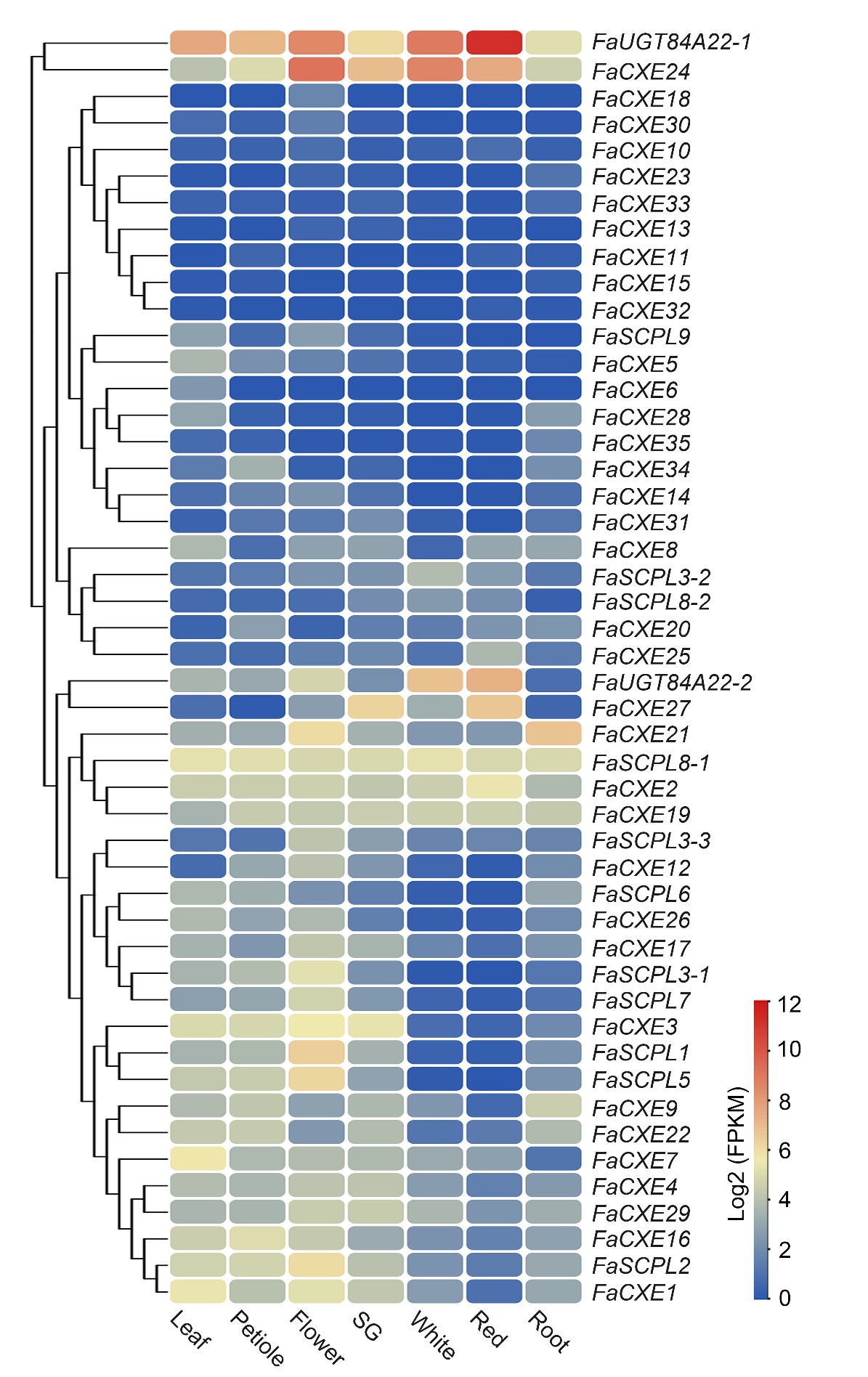
~~**Fig. S4 Expression patterns of *FaSCPL-AT*, *FaUGT84A22*, and *FaCXE* genes in different strawberry tissues**


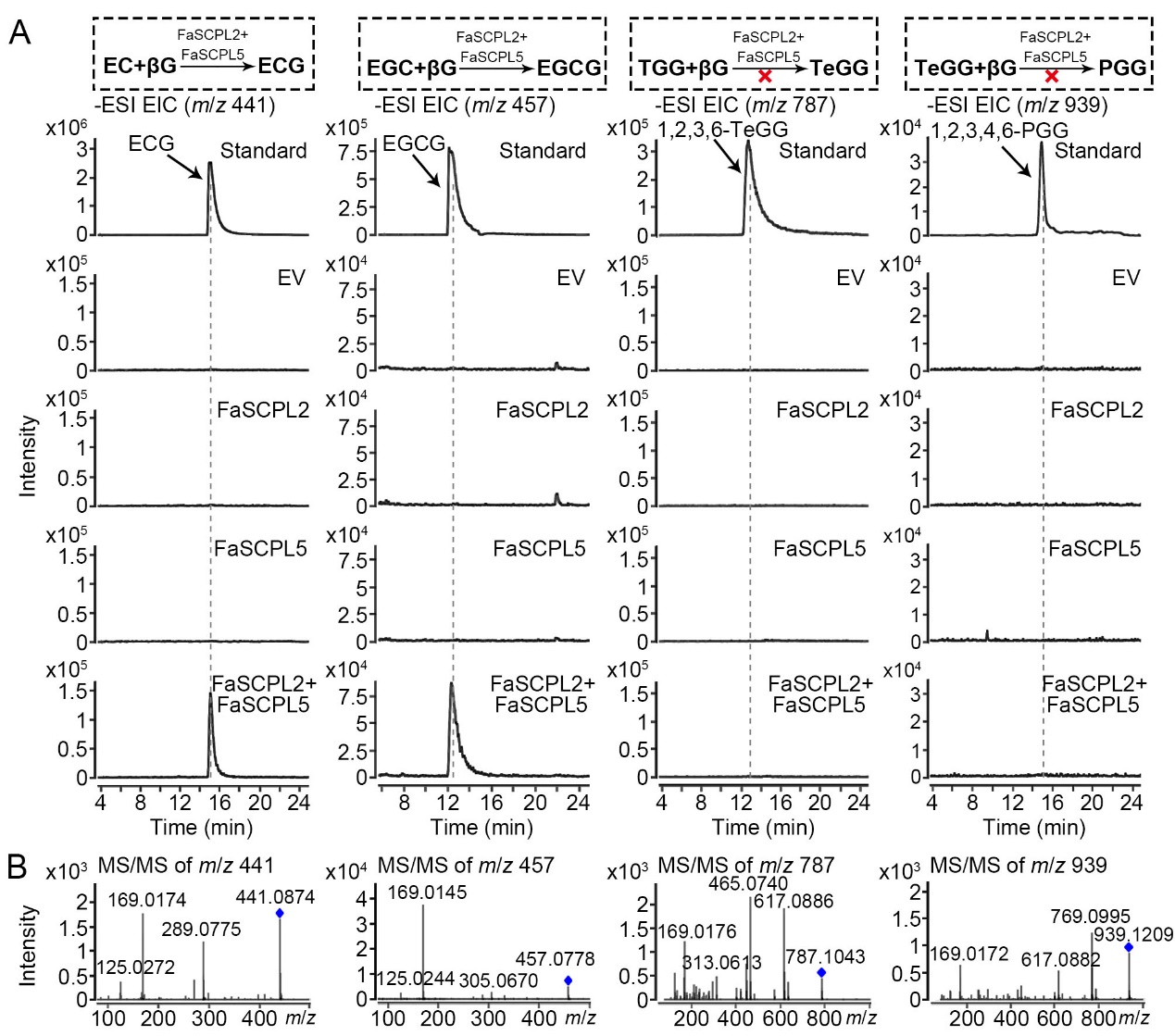


**Fig. S5 Enzyme activity analysis of FaSCPL2 and FaSCPL5**

A, The extraction ion chromatogram (EIC) of enzymatic products of transient expression of *FaSCPL2* and *FaSCPL5* in *N. benthamiana* using the following sets of substrates: EC and βG, EGC and βG, 1,3,6-TGG and βG, 1,2,3,6-TeGG, and βG from left to right. B, MS/MS profiles information for ions *m*/*z* 441 (epicatechin gallate, ECG), *m*/*z* 457 (epigallocatechin gallate, EGCG), *m*/*z* 787 (1,2,3,6-TeGG), and *m*/*z* 939 (1,2,3,4,6-PGG) from left to right.


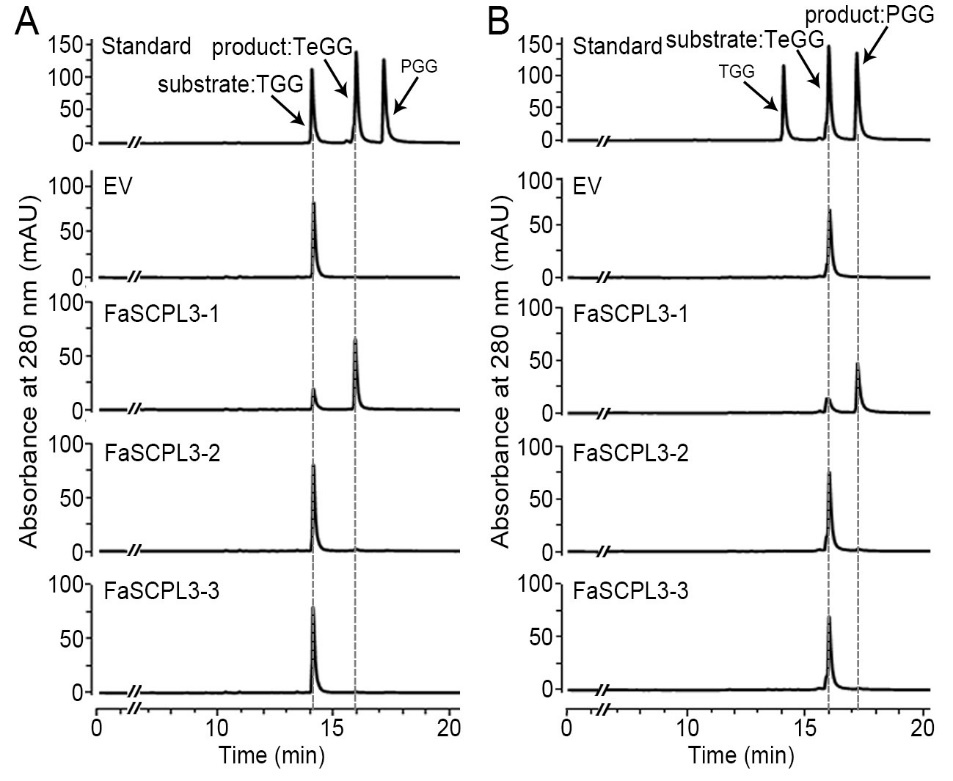


**Fig. S6 Enzyme activity analysis of FaSCPL3s**

A and B, UPLC analysis of the enzymatic products of *FaSCPL3s* transiently expressed in *N. benthamiana* using the following sets of substrates: 1,3,6-TGG and βG (left), 1,2,3,6-TeGG, and βG (right), respectively.


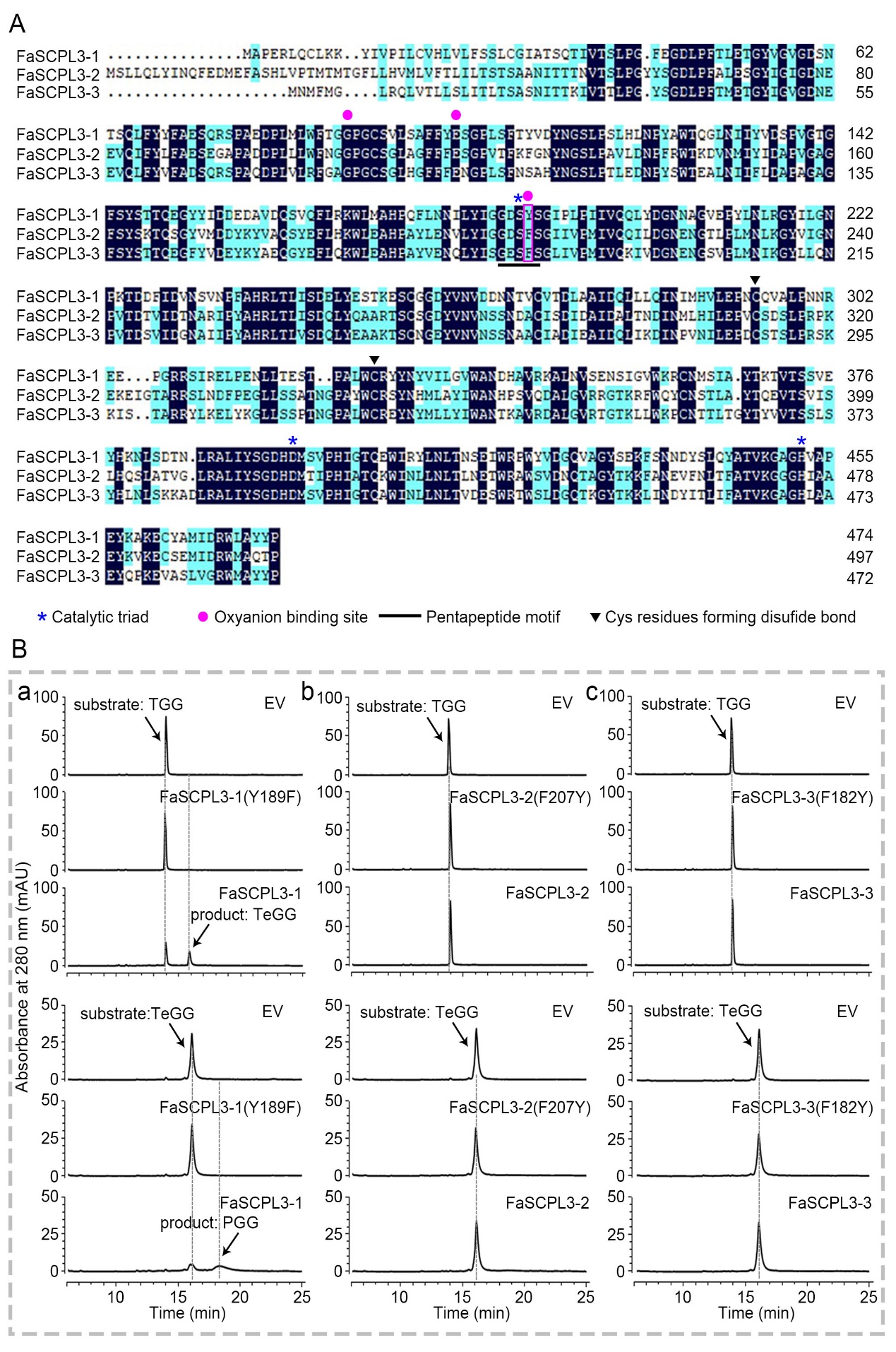


**Fig. S7 Enzyme activity analysis of the site-directed mutations in FaSCPL3s**

A, Amino acid alignment of the FaSCPL3 proteins. Blue asterisks indicate catalytic triads, pink dots denote oxyanion-binding sites, a black line represents pentapeptide motif and a black inverted triangle indicates Cys residues forming disulfide bonds. B, UPLC analysis of enzymatic products of site-directed mutation of FaSCPL3s. a-c, Effects of amino acids point mutation FaSCPL3-1 (left), FaSCPL3-2 (middle), FaSCPL3-3 (right) using the following sets of substrates: 1,3,6-TGG, and βG (top panel), 1,2,3,6-TeGG, and βG (bottom panel), respectively.


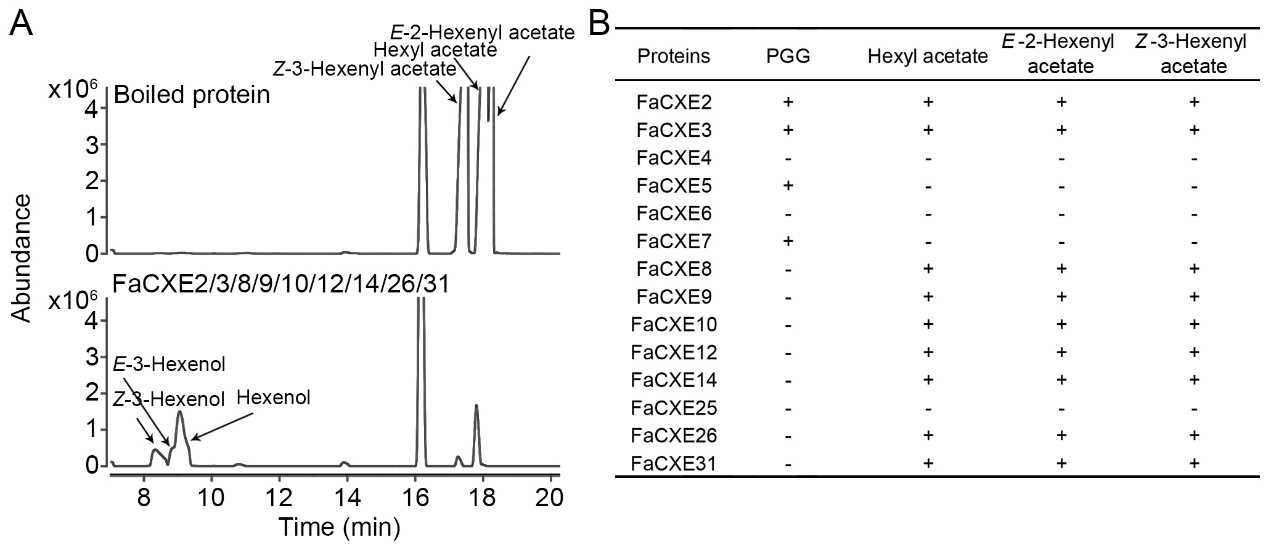


**Fig. S8 Enzymatic assays for recombinant FaCXE proteins**

A, Catalytic activity of recombinant FaCXE proteins using hexyl acetate, *Z*-3-hexenyl acetate, and *E*-2-hexenyl acetate as substrates. B, Summary results of enzymatic assays of recombinant FaCXEs using PGG and volatile esters as substrates.


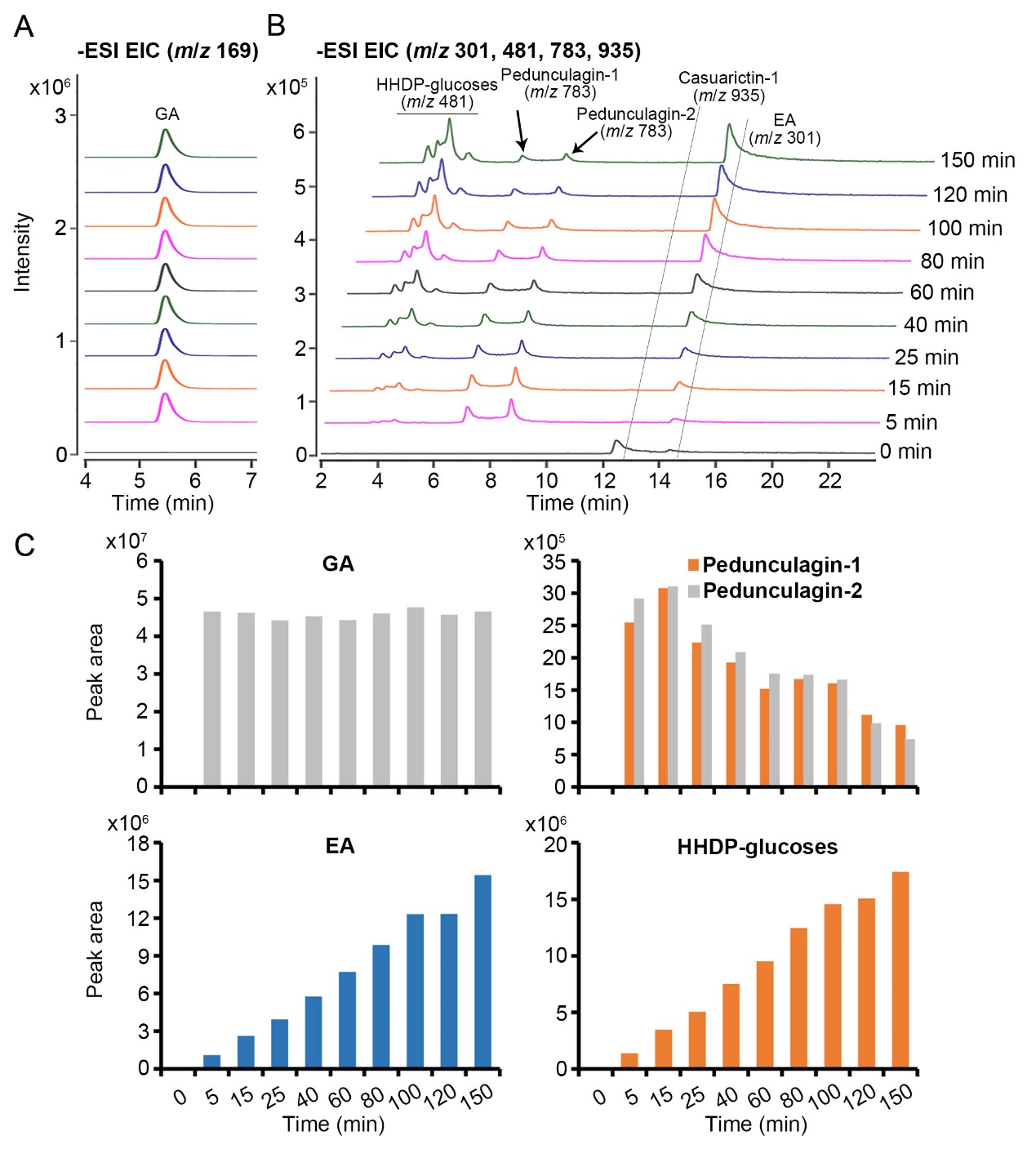


**Fig. S9 Enzyme activity analysis of recombinant FaCXE proteins using casuarictin-1 hydrolytic assay**

A, Analysis of GA in the time gradient of recombinant protein FaCXEs hydrolyzing casuarictin-1. B, Analysis of intermediate products in the time gradient of the reaction of recombinant protein FaCXEs. C, Relative quantitative analysis of products in the time gradient of the hydrolysis reaction.


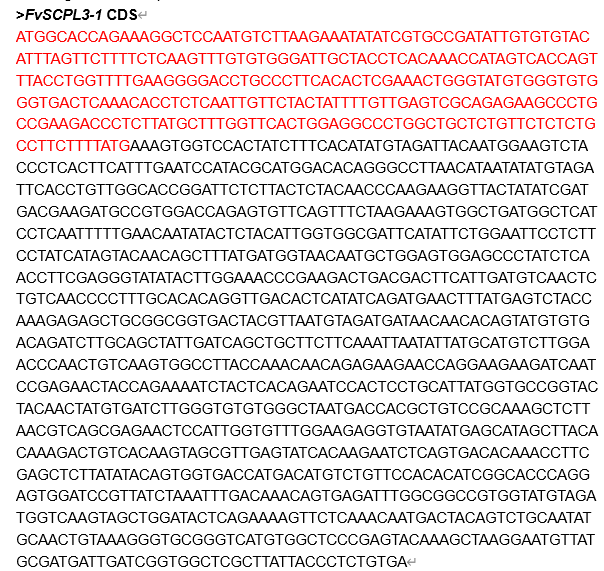


**Fig. S10 The DNA sequence of *FvSCPL3-1***

The DNA sequence selected for the RNAi construct to downregulate the expression of *FvSCPL3-1* is marked in red.


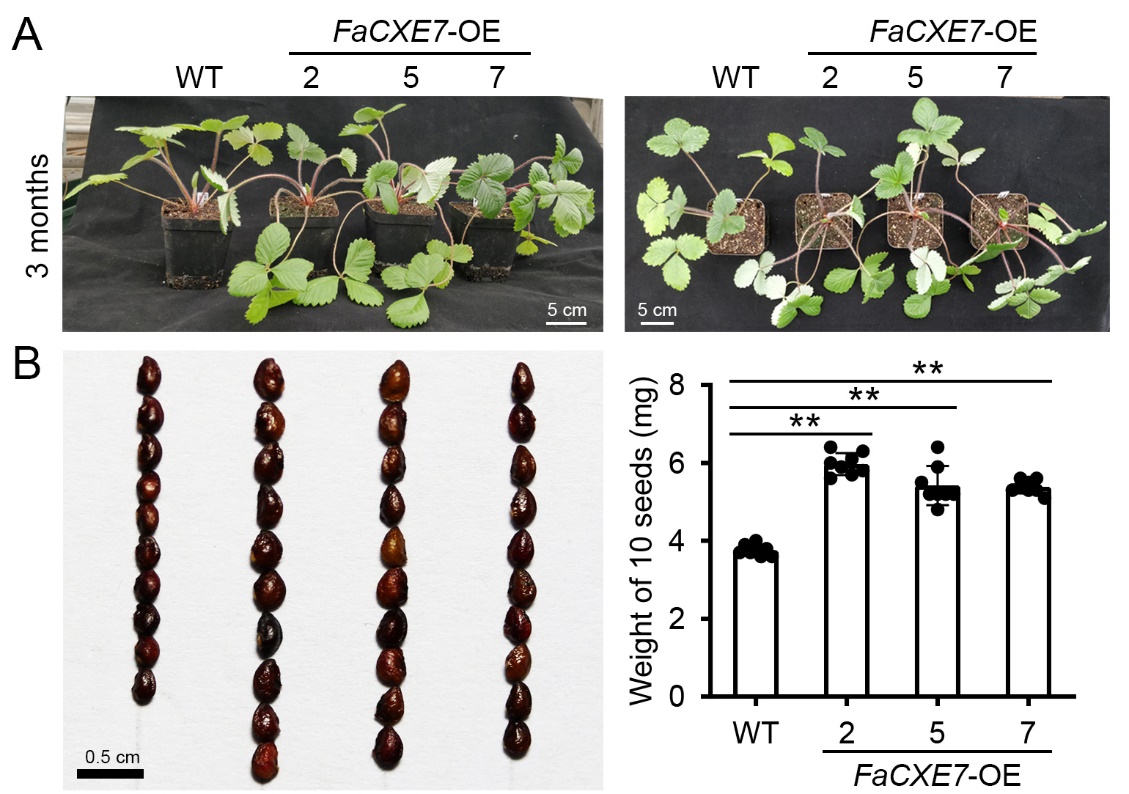


**Fig. S11 Phenotypes of the transgenic strawberry overexpressing *FaCXE7***

A, Phenotypes of WT and overexpression lines after three months of growth. Scale bar: 5 cm. B, Seed phenotypes and weights of the WT and overexpression lines after harvest. Scale bar: 0.5 cm. Data were presented as mean ± SE of three biological replicates. Statistical significance was analyzed based on Student’s *t*-test: **P*<0.05, ***P* <0.01.


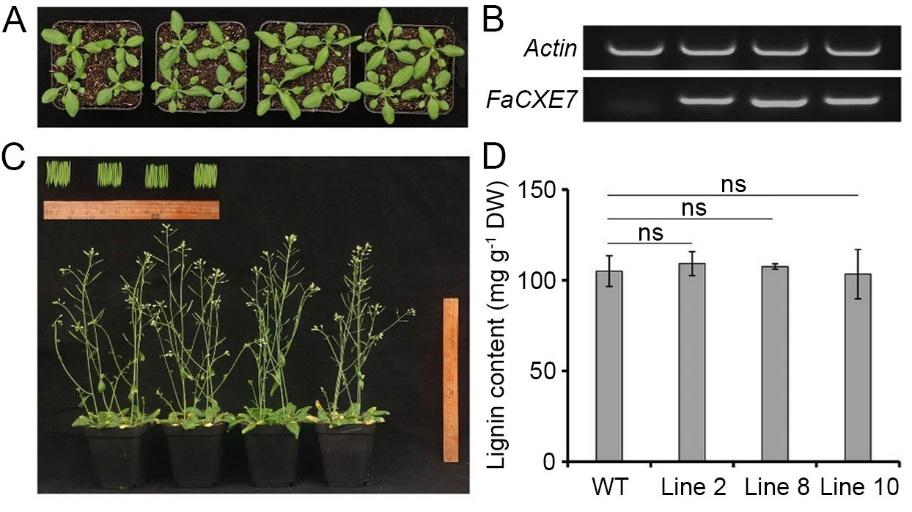


**Fig. S12 Overexpression of *FaCXE7* in *Arabidopsis***

A, Phenotypes of WT and transgenic *Arabidopsis* overexpressing *FaCXE7*. B, Semiquantitative RT-PCR analysis of *FaCXE7* and the housekeeping gene *AtActin in* WT and transgenic lines. C, Phenotypes of WT and transgenic lines during flowering. C, Lignin content of the WT and transgenic lines.


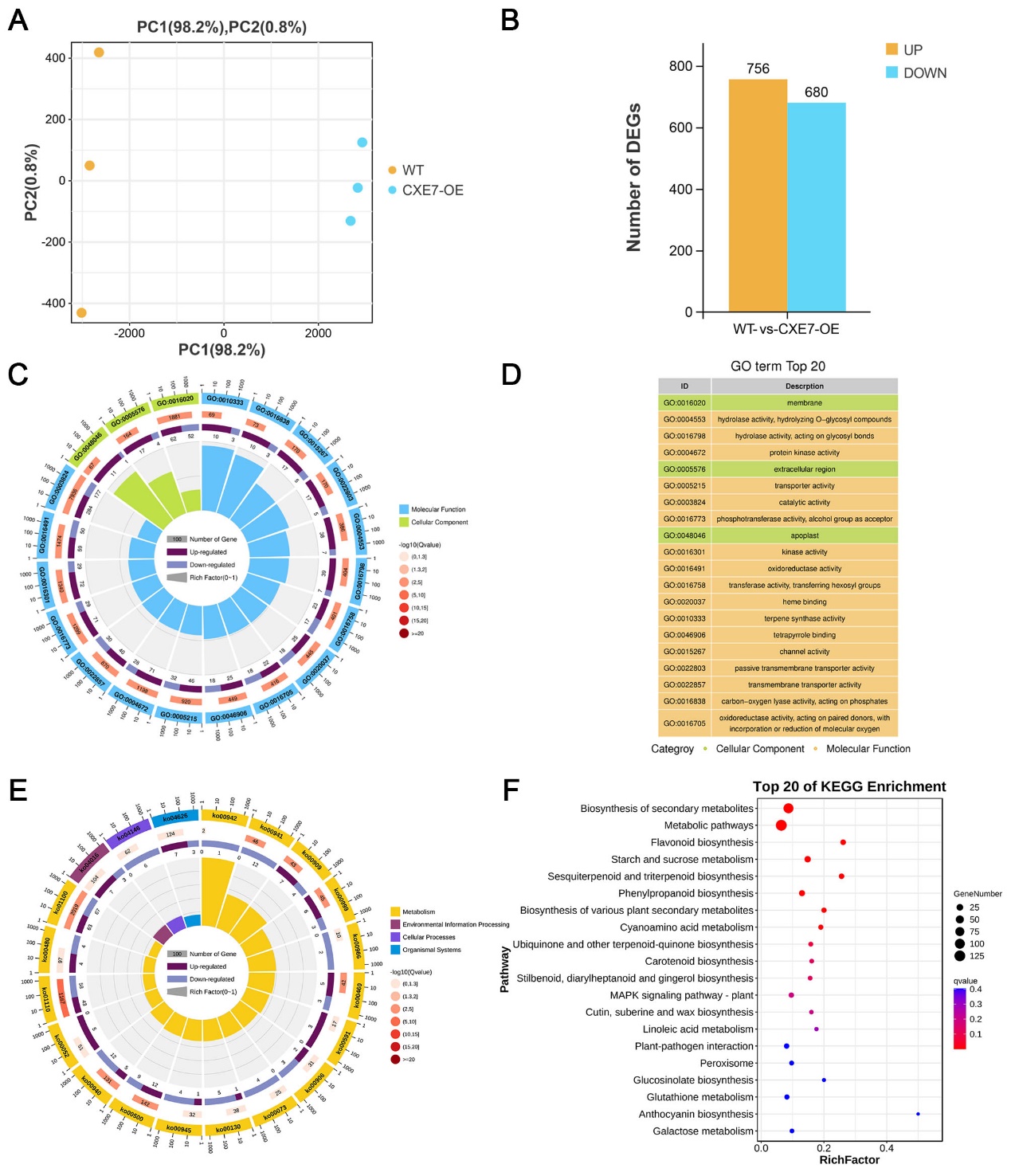


**Fig. S13 Transcriptome analysis of the transgenic strawberry overexpressing *FaCXE7***

A, Principal Component Analysis (PCA) of transcriptome data. B, Number of upregulated and downregulated DEGs in WT and *FvCXE7*-OE strawberry plants. C, GO enrichment analysis of DEGs. The first circle outside represents the top 20 enriched GO terms, and outside the circle is the coordinate scale of the number of differential genes. Different colors represent diverse ontologies. The second circle denotes the number of genes and *Q* values in each GO term. The third circle signifies the bar chart of the upregulated and downregulated DEG proportions. The fourth circle indicates the rich factor value of each GO term. D, The list of top 20 GO terms. Different colors represent varying ontologies. E, Circle diagram of KEGG enrichment analysis. The first circle indicates the top 20 enriched pathways, and outside the circle is the coordinate scale of the number of differential genes. Different colors represent various A classes. Yellow denotes metabolism, dark purple stands for environmental information processing, light purple signifies cellular processes, and blue represents organismal systems. The second circle indicates the number of genes and *Q* values in each pathway, the length of the bar denotes the number of genes, and the colors represent *Q* values. The third circle illustrates the bar chart of the upregulated and downregulated DEG proportions. Dark purple denotes upregulated genes and light purple represents downregulated genes. The fourth circle indicates the rich factor value of each KEGG term (the ratio of the number of DEGs/the number of genes in this pathway). The background grid scale for each grid is 0.1. F, Bubble chart of KEGG enrichment analysis. The top 20 KEGG pathways are listed with *Q* values. The size of the dots indicates the gene number, and the colors represent the *Q* values.
